# Integrating selective signature analysis and genome-wide association study to identify candidate genes associated with litter size in Leizhou goat

**DOI:** 10.5713/ab.260280

**Published:** 2026-07-27

**Authors:** Da Feng, Siyi Hu, Jian Ma, Chunmei Du, Guangxian Zhou, Chen Wei, Shangquan Gan

**Affiliations:** 1 College of Coastal Agricultural Sciences, Guangdong Ocean University, Zhanjiang, China

**Keywords:** Candidate Genes, Genome-wide Association Study, Leizhou Goat, Litter Size, Selection Signature Analysis

## Abstract

**Objective:**

Litter size is a key trait for evaluating goat reproductive performance, since it has a direct influence on the breeding efficiency and production cycle profitability. However, a comprehensive understanding of its genetic architecture, including the causative mutations and their functional impacts, remains scarce, hindering its application in precision breeding.

**Methods:**

A total of 92 female Leizhou goats were genotyped through whole-genome sequencing, and their litter sizes were measured over three consecutive births. Following genotype and phenotype data collection, population genetic structure analysis, selection signature analysis, and genome-wide association study (GWAS) were performed.

**Results:**

We identified 20,802,514 SNPs in the 92 Leizhou goats. Analysis of genetic structure revealed that the high-litter-size group exhibited greater genetic diversity than the low-litter-size group, although no significant genetic differentiation was found between them. We identified 340 putative selective regions and 346 candidate genes across the genome. A total of 37 candidate genes were identified as being closely associated with reproductive traits, including *BMP6*, *MAPK8*, *RPTOR*, etc. The GWAS revealed 63 quantitative trait loci (QTLs) at suggestive significance, 52 of which were associated with litter size. After that, 207 candidate genes were annotated, and four of them were highlighted that have been previously reported to be associated with reproductive processes (e.g., *TMEM100*, *PDGFD*, *NLRP9*, and *ARHGAP22*). By integrating the results from both approaches, we identified *MAPK8* as a potential candidate gene associated with litter size. Furthermore, our findings from motif recognition, transcription factor prediction, and previous studies lead us to propose that the *BMP6-GATA4-MAPK8* signaling pathway may play a key role in regulating litter size in goats.

**Conclusion:**

Our findings identify several candidate genes and putative associated variants for litter size, providing valuable insights into the genetic basis of reproductive traits in goats.

## INTRODUCTION

Goats were domesticated almost 10,500 years ago in the Fertile Crescent and subsequently dispersed globally through human migrations [1,2]. Under the influence of natural and artificial selection, they have developed distinct breeds adapted to various regional environments [3]. At the southernmost tip of mainland China, the Leizhou Peninsula is characterized by a tropical monsoon climate with year-round high temperatures and humidity. This environment has shaped the Leizhou goat, a local breed renowned for its tolerance to heat and humidity [4]. The meat of Leizhou goats is known for its delicious, and even fat distribution, with minimal muttony smell. Their hides are compact, lightweight, and exhibit good elasticity. Furthermore, Leizhou goats demonstrate strong reproductive performance, reaching sexual maturity at approximately four months of age and exhibiting non-seasonal estrus. Throughout the long-term artificial selection of Leizhou goats, two distinct body-type have variants gradually emerged: the long-legged type and the short-legged type. Long-legged goats are generally larger in body size and tend to produce singletons. In contrast, short-legged goats are smaller but exhibit higher litter size, frequently giving birth to twins or triplets [5]. Historically, local herders have favored short-legged does for their greater reproductive output, implying that litter size may have been an indirect target of selection. Despite these valuable traits, the breeding system for the Leizhou goat is not yet fully established, which hinders the full exploitation of its reproductive potential [6]. This limitation not only restricts the further enhancement of the breed’s advantages but also increases the risk of population degradation, including reduced genetic diversity and declining production performance [7,8]. Therefore, enhancing the litter size of Leizhou goats is vital for both securing this breed’s future and boosting global goat productivity to meet rising demand.

Litter size is a key reproductive performance in goats. It is typically a low-heritability, polygenic trait (0.08–0.18), with numerous genes each exerting small, additive effects [9,10]. The genetic study of this trait presents several challenges. First, phenotypic variation is limited, particularly in goats, where triplets or larger litters are of low frequency. Second, it is influenced by complex physiological processes, including folliculogenesis, hormonal regulation, and early embryonic development [11]. These factors constrain genetic improvement through traditional selection. Fortunately, advances in and reduced costs of sequencing technologies have established whole-genome sequencing (WGS) as a powerful, mainstream approach for identifying the genomic variants underlying complex traits, thereby providing new avenues to address these limitations [12].

The genome encompasses an organism’s complete set of genes, along with their regulatory and non-coding regions, forming its fundamental genetic blueprint. Thus, genomic sequencing serves as a powerful tool for elucidating genetic diversity, population structure, and adaptive evolution, as well as for identifying variants underlying complex traits [13,14]. In recent years, WGS has been widely applied to investigate the genetic basis of reproductive traits in local goat breeds [15,16]. Studies have demonstrated that genes regulating reproductive traits often undergo rapid divergence, a phenomenon generally regarded as a hallmark of adaptive evolution [17]. Leizhou goats are known for their remarkable adaptability to high-temperature environments and exhibit strong reproductive performance. Therefore, we hypothesize that this breed may harbor unique genetic variants in reproduction‐related genes. Consequently, identifying candidate genes associated with litter size is of great significance for elucidating the underlying genetic basis of its reproductive traits. However, the genetic architecture and mechanisms underlying the reproductive traits of Leizhou goats remain poorly understood. Although transcriptomic studies have identified several candidate reproduction-related genes [6,18], the inherent limitations of such approaches preclude a comprehensive understanding of the underlying genetic basis and regulatory networks.

Accordingly, this study utilized WGS to: (1) assess the genetic diversity and population structure of Leizhou goats with high and low litter sizes; (2) identify candidate genes associated with litter size through selective sweep analysis and genome-wide association study (GWAS); and (3) discover enriched biological processes and functional terms involved in litter size. Our findings elucidate the genetic architecture of litter size in Leizhou goats and provide a theoretical foundation for developing targeted breeding strategies.

## MATERIALS AND METHODS

### Sample preparation and sequencing

In this study, blood samples were collected from 92 Leizhou goats at a conservation farm (Zhanjiang, Guangdong Province) for WGS. All goats belonged to the same generation and were of similar age. They were raised under identical housing conditions, with one ram allocated per pen to allow for natural mating. The high- and low-litter-size (LL) groups originated from the same breeding population and management environment, ensuring a balanced sire distribution and equal mating opportunities for all ewes. We collected data on litter size over three consecutive parities for these individuals and divided them into a high-litter-size (HL) group (mean litter size≥2) and a LL group (mean litter size<2) according to their average litter size. The phenotypic records of litter size for these individual goats are presented in Supplement 1. The 92 Leizhou goats in this study include the 20 used in a previous study by the research group [19].

This study performed genetic sequencing on 92 Leizhou goats. The collected jugular vein blood was initially stored at −80°C. Subsequently, sample testing was performed to assess concentration, sample integrity, and purity. Sequencing was conducted on the DNBSEQ-T7 platform, yielding 150 bp paired-end reads, with 20 individuals sequenced at an average depth of 11.32X and the remaining 72 individuals at an average depth of 1.06X.

### Mapping and variant calling

Quality control of the raw resequencing data was performed with Fastp (v0.23.4), and the clean reads were aligned using BWA (v0.7.17) with the ARS1 (GCA_001704415.1) reference genome. SAMtools (v1.21) was used to sort and mark duplicates for the aligned files. The GATK HaplotypeCaller tool was used to call all variants and store them in a gVCF file. The single nucleotide polymorphism (SNP) loci were extracted for the gVCF file using GATK’s SelectVariants command, followed by quality control using the VariantFiltration command, with parameters set to QD<2.0, MQ<40.0, FS>60.0, SOR>3.0, MQRankSum<−12.5, ReadPosRankSum<−8.0.

Genotype imputation for the 72 low-coverage sequenced individuals was performed using GLIMPSE2 (v2.0.0) [20]. We utilized the multi-breed Goat Genomics Reference Panel, comprising 2,105 goats and developed by the Ruminant Genetics and Evolution team at Northwest A&F University (https://animal.nwsuaf.edu.cn/). The average sequencing depth of this reference panel is 10×. This imputation achieved a high average concordance rate of 98%. This high-fidelity imputation effectively mitigates the inherent limitations of low-coverage sequencing and ensures the validity of our subsequent inferences. The imputed data were then merged with the genotype data of the remaining 20 goats using BCFtools (v1.10.2). Subsequently, further filtering was performed using VCFtools (v0.1.14) to retain bi-allelic loci (–min-alleles 2 –max-alleles 2), remove loci with a minor allele frequency (MAF) less than 0.01 (−maf 0.01), and exclude loci with a Hardy-Weinberg equilibrium (HWE) p-value of less than 1×10^−6^ (−hwe 0.000001). Finally, a total of 20,802,514 SNPs in the reference population were kept for further study.

### Population structure

As both principal component analysis (PCA) can be significantly influenced by sample size, it used balanced sample sizes across populations to reduce potential biases [21]. Thirty individuals were randomly selected from LL individuals to maintain a population size comparable to that of the HL groups. Consequently, this resulted in a final dataset of 60 individuals (30 HL and 30 LL) for all population structure analyses. The first two Principal Components were calculated using Plink (v1.9.0). Additionally, a neighbor-joining (NJ) tree was constructed with Phylip (v3.697) based on genome-wide SNPs to illustrate the phylogenetic relationships among the individuals.

### Genetic diversity

Genetic diversity indices, including expected heterozygosity (H_E_), observed heterozygosity (H_O_), MAF, and the inbreeding coefficient based on heterozygosity (F_IS_), were calculated using Plink (v1.9.0) with the parameters --hardy, --freq, and --het, respectively. The observed number of alleles (A), effective number of alleles (Ae), and polymorphism information content (PIC) were subsequently derived from the allele frequency data. Linkage disequilibrium (LD) decay analysis was performed using PopLDdecay (v3.41) with the parameters -MaxDist 500 -MAF 0.005 -Het 0.9 -Miss 0.25. The resulting plot was generated using the script Plot_MultiPop.pl with the options -bin1 10 -break 1000 -bin2 200 -KeepR.

The runs of homozygosity (ROH) were identified by applying the following criteria in Plink (v1.90): a minimum of 50 SNPs in homozygosity (–homozyg-window-snp); a maximum value of 100 kb for the gap between two consecutive SNP markers (–homozyg-gap); 1 heterozygous locus in the ROH segment (–homozyg-window-het); homozygous length greater than 250 Kb (–homozyg-kb), and no more than 1 missing genotypes across the animals (–homozyg window-missing). The sum of ROH segments per goat was calculated considering four classes in megabases (Mb): between 1 and 2 Mb, 2 and 4 Mb, 4 and 8 Mb and higher than 8 Mb. Finally, the genomic inbreeding coefficient (F_ROH_) was calculated for each individual as the total length of all ROH segments divided by the total length of the autosomes covered by the SNP array: $F_{ROH}=\frac{\sum_{k}legnth\left(ROH_{k}\right)}{L}$ [22].

### Genome-wide selection signature analysis

To detect genome-wide selective sweeps associated with litter size, the genome-wide assignment of fixation index (*F*_ST_) values and hp ratios were calculated for these defined group pairs (VCFtools v0.1.14). Average SNP *F*_ST_ values were plotted in 20 kb genomic bins with a 5 kb step. Nucleotide diversity (π) was estimated for the same bins (VCFtools v0.1.14). The *F*_ST_ values were Z-transformed as follows: *Z*(*F_ST_*) = (*F_ST_*−*μF_ST_*)/*σF_ST_*, in which *μF_ST_* is the mean *F_ST_* and *σF_ST_* is the standard deviation of *F_ST_*. The π ratios were log2-transformed. Then we evaluated and arranged the empirical percentiles of Z(*F*_ST_) and log2(π ratio). Genomic regions under selection were defined as those falling within the top 5% of both the Z(*F*_ST_) and log2(π ratio) distributions.

Additionally, we used the Selscan (v1.2.0) to computed the cross-population extended haplotype homozygosity (XP-EHH) for each SNP between the high and low litter size groups, averaging the values in non-overlapping 20 kb windows. Windows with XP-EHH values in the top 5% were considered candidate selective sweeps. Candidate genes under selection were defined as those overlapped by sweep regions. Two additional statistics, the Tajima’s D and Composite Likelihood Ratio (CLR) test, were applied with a 20-kb sliding window and a 5-kb step size to confirm the top signals.

### Genome-wide association study

To locate the regions identified by the selective sweep analysis and identify direct genotype-phenotype associations, we performed a GWAS for litter size. The analysis was based on a panel of 92 Leizhou goats and implemented using a mixed linear model (MLM) in GEMMA (v0.96) [23]. The model accounted for population stratification by including the top PCs derived from genome-wide SNPs as fixed effects. A genetic relatedness matrix was fitted as a random effect to control for spurious associations due to familial relatedness. Additionally, fixed effects such as the age of the ewe and the farm were included as covariates in the model. The statistical model applied was as follows:


(1) y=Wα+Xβ+Zu+e

where *y* the vector of phenotypes for litter size traits, *α* is the fixed effect of the SNP tested for association, *W* is a matrix containing SNPs; *β* is the vector of the fixed effects, including year, farm and the first 5 principal components, *X* is the incidence matrices assigning observations to fixed effects; *u* is the vector of the remaining polygenic effect with $u∼N(0,G\sigma_{u}^{2})$, where *G* is the genomic-based relationship matrix (GRM) calculated as $G=\frac{MM}{2\sum p_{i}\left(1-p_{i}\right)}$; *M* is the centered genotype matrix, *p_i_* is the allele frequency at *i*-th SNP, and $\sigma_{u}^{2}$ is the additive genetic variance of polygenic effects; *Z* is the incidence matrix for *u*; and *e* is a vector of residual effects with $e∼N\backslash \text{left}(0,\text{I}\sigma_{e}^{2})$, where $\sigma_{e}^{2}$ is an identity matrix and is the residual variance.

To determine the significance threshold for the GWAS, Plink (v1.9.0) was used to perform LD filtering with the command --indep-pairwise 50 5 0.05. Based on 6,080,395 independent SNPs, we defined SNPs as a large effect SNP and a small effect SNP with a p<5×10^−8^ and p<1×10^−5^, respectively. SNPs with a distance greater than 1 Mb were considered to belong to two different quantitative trait locis (QTLs). The SNP with the most significant p-value within each QTL is defined as the Lead SNP. To conduct sensitivity analysis, we additionally performed a GWAS using logistic regression models in the PLINK (v2.0.0) software.

### Functional enrichment analysis

To investigate the putative biological functions of protein-coding genes located within the selective sweep regions and QTL intervals, we performed Kyoto Encyclopedia of Genes and Genomes (KEGG) pathway enrichment analysis. The analysis was conducted using the KOBAS 3.0 server (http://bioinfo.org/kobas/), with the goat protein sequences of the target genes as input and the complete goat genome as the background set. Overrepresented pathways were identified using the hypergeometric test, and a corrected p-value of <0.05 was considered statistically significant.

### Phenome-wide association study of candidate genes in humans

To explore whether the orthologues of candidate genes detected for litter size traits in goat were associated with complex traits in humans, we conducted a Phenome-wide association study (PheWAS) for each of the candidate genes based on the human GWAS data in the GWASATLAS database (https://atlas.ctglab.nl/PheWAS). Briefly, we explored the GWAS summary statistics of 465 complex phenotypes from 101 human GWAS. For visualization, we classified these complex traits into 24 trait domains.

### Verification of significant variation in goat populations

To contextualize our findings within a broader genetic background, we compared the allele frequencies of potential candidate variants between established high- and low-reproduction goat breeds. Based on previous literature, Jintang Black (JTBG, n = 30), Dazu Black (DZBG, n = 30), Macheng Black (MCBG, n = 27), and Longlin goats (LLG, n = 31) were classified as high-reproduction breeds, whereas Yunling Black (YLBG, n = 30), Yimeng Black (YMBG, n = 30), and Lüliang Black goats (LLBG, n = 30) were classified as low-reproduction breeds. For the significant loci, we extracted the genotype data and the corresponding litter size phenotype data, and used the Wilcoxon test to assess whether there were significant phenotypic differences among the different genotypes.

### Prediction of motifs and transcription factors in gene promoter regions

We analyzed the promoter sequences of these candidate genes to identify potential transcription factor binding sites. Sequence motifs were predicted de novo using the MEME suite (https://memesuite.org/meme/tools/meme) [24]. The identified motifs were then compared against known motif databases using the Tomtom tool within the MEME suite. Significantly matched motifs (p-value<0.05) were used to query the JASPAR database (https://jaspar.elixir.no/) to predict the corresponding transcription factors.

### Quantification and differential expression analysis of candidate gene in ovary and pituitary tissues

To validate the effects of candidate genes on reproductive traits, transcriptomic data from 20 Yunshang black goats (ovarian tissues, accession number PRJNA726268) and 7 Leizhou goats (pituitary tissues, accession number PRJNA 1043736) with high and low fecundity were retrieved from the NCBI Sequence Read Archive (SRA). The raw reads were subjected to quality control using fastp (v1.1.0). Subsequently, the resulting clean reads were aligned to the reference genome using STAR (v2.7.11). Transcript quantification was then performed utilizing StringTie (v3.0.3) and featureCounts (v2.1.1).

## RESULTS

### Sequencing variants in high and low litter size populations of Leizhou goats

We performed whole-genome resequencing on 92 Leizhou goats (20 at high depth and 72 at low depth with subsequent imputation). After merging and quality control, we obtained a final set of 20,802,514 high-quality autosomal SNPs from all 92 individuals. Genomic distribution analysis revealed that the vast majority of SNPs (19.38 million, 93.16%) were in intronic and intergenic regions, whereas only 0.14 million (0.68%) were exonic (Supplements 2, 3B). Among these exonic SNPs, 88,132 were synonymous, and 52,621 were non-synonymous (Supplements 3C, 4). These SNPs were uniformly distributed across all autosomes (Supplement 3A).

### Population structure and genetic diversity of Leizhou goat

Using the WGS data from Leizhou goats described above, we aimed to assess the genetic and evolutionary relationships associated with divergent litter sizes. Based on differences in average litter size, we classified Leizhou goats into two phenotypic groups: HL group and LL group.

Population genetic analysis revealed distinctions between the HL and LL groups. The LD decay patterns of the HL and LL groups did not show obvious differences (Figure 1A). PCA showed no clear population stratification, with the first two principal components (PC1 and PC2) explaining 36% and 17.2% of the variance, respectively (Figure 1B). This pattern minimizes the risk of spurious associations in subsequent analyses. A NJ phylogenetic tree based on genome-wide SNPs showed extensive mixing between the HL and LL groups, although some branches exhibited an asymmetric distribution of individuals from the two groups (Figure 1C).

The HL group exhibited higher values for key genetic diversity indices, including H_O_, H_E_, PIC, and nucleotide diversity (Table 1). Among these, the nucleotide diversity of the HL group was highly significantly higher than that of the LL group (p<0.01) (Figure 1D). Additionally, we counted the number of ROH detected in the HL and LL groups. The number of ROHs detected in the HL group was significantly lower than that in the LL group (Figure 1E), which also indicates that the HL group has a higher level of genetic diversity. We calculated the inbreeding coefficients based on heterozygosity and ROH, respectively. However, the statistical test results showed that the inbreeding coefficients between the two groups were not significantly different (Figure 1F and Table 1).

### Selection region and genes related to litter size in Leizhou goat

To identify selective sweeps associated with the litter size in Leizhou goats, we compared the HL and LL populations using 20-kb sliding windows across the genome. We estimated *F*_ST_, differences in nucleotide diversity (log2π-ratio HL/LL), and XP-EHH (Figure 2A). Next, using the top 5% cutoffs for *F*_ST_, π-ratio, and XP-EHH, we defined the windows that met all three statistical thresholds (*F*_ST_>0.03, log2π-ratio>0.3, and XP-EHH>1.41) as putative selective windows, and ultimately obtained 1,492 windows that showed significant signals of selection (Figure 2B). After merging adjacent candidate windows, we identified 340 unique selective sweep regions encompassing 346 protein-coding genes (Supplement 5). Notable genes within these regions have established roles in reproduction (e.g., *BMP6*, *B3GALT5*, *NCKAP5*, *SPAG17*), growth (e.g., *CNOT2*, *RAB6B*, *ADGRL2*), and heat stress response (e.g., *HSPA12B*, *ADGRB1*, *GRIA4*) (Supplement 5).

Functional enrichment analysis of the candidate genes identified significant associations (p<0.05) with 38 signaling pathways (Supplement 6). Notably, several key genes were enriched in pathways critical for reproduction: three genes (*BMP6*, *INSR*, and *ADCY3*) were involved in the ovarian steroidogenesis pathway, and four genes (*ANAPC1*, *CDC25B*, *MAPK8*, and *ADCY3*) participated in the progesterone-mediated oocyte maturation pathway (Figure 2C). Both pathways play essential roles in regulating oocyte maturation and follicular development, which are key biological processes directly determining litter size in goats.

We identified a genomic region encompassing the *BMP6* gene—a participant in the ovarian steroidogenesis pathway—that exhibited strong signatures of selection (*F*_ST_ = 0.095, log2(π-ratio) = 0.827, XP-EHH = 4.47) (Supplement 7). This signal was further supported by a large negative Tajima’s D value and a high CLR score, collectively indicating strong positive selection at this locus in Leizhou goats (Figure 2D). To validate whether the *BMP6* gene region was influenced by the chosen random subset, we conducted a selection signal analysis using the full set of 92 individuals. The results indicated that the *BMP6* gene region consistently showed strong selection signals (Supplement 8). Analysis of the haplotype structure in this region revealed distributional differences between the HL and LL groups (Figure 2E). To identify the potential candidate variant, we compared allele frequencies of all SNPs within the *BMP6* region and identified three SNPs (rs7458736596, rs7458763602, rs646562093) with significantly higher alternative allele frequencies in the HL group (0.60, 0.55, 0.62) than in the LL group (0.30, 0.20, 0.25) (Figure 2F). This significant disparity suggests their potential role in influencing litter size. Subsequent genotype analysis showed that the frequency of homozygous mutant genotypes for two of these SNPs (rs7458763602, rs646562093) was significantly elevated in the HL group, while the difference for the third SNP (rs7458736596) was less pronounced (Figure 2G). Furthermore, we validated these loci using additional independent high- and low-fertility groups (High: JTBG, DZBG, MCBG, LLG, n = 118 vs. Low: YLBG, YMBG, LLBG, n = 90). For loci rs7458763602 and rs646562093, significant phenotypic differences were observed between the mutant (AG) and wild-type (GG) genotypes, with p-values of 0.039 and 0.018, respectively (Supplement 9). Intriguingly, the alternative alleles of these candidate SNPs, which were at relatively high frequencies in the Leizhou goats, were found at much lower frequencies in these external validation cohorts, suggesting they may represent population-specific variations.

### Genome-wide association study revealed variation and gene responsible for litter size in Leizhou goat

We performed a GWAS for litter size in 92 Leizhou goats using a linear mixed model to identify genetic loci with high confidence. Q-Q plots of the GWAS are presented in Supplement 10. As a sensitivity analysis, we performed a binary GWAS using a generalized linear model (GLM). The analysis identified fewer significant SNPs compared with the MLM approach (Supplement 11). Additionally, the QQ plot exhibited a slight deflation in the tail region (genomic inflation factor λ = 1.21) (Supplement 12), which is likely attributable to the reduced statistical power resulting from phenotype dichotomization. Given that the MLM approach detected consistent signals while the binary analysis exhibited a loss of power, we primarily focused our interpretations on the MLM results.

We identified 264 SNPs that were suggestively associated (p<1×10^−5^) with litter size. Candidate region annotation of these signals defined 63 independent QTLs, and their lead SNPs (Figure 3A). Annotation of these QTLs identified 207 protein-coding genes. These include candidates with previously reported roles in reproduction (e.g., *PDGFD*, *NLRP9*, *ARHGAP22*), muscle development (e.g., *GRB10*, *SOX13*, *MYOM2*), and body size (e.g., *PPP2R2B*, *N4BP3*, *PRDM6*) (Supplement 13). The genomic inflation factor (λ) was 0.998, indicating effective control for population stratification. Functional enrichment analysis revealed that these genes were significantly overrepresented (p<0.05) in 14 signaling pathways, including the MAPK, dopaminergic synapse, and oxytocin signaling pathways (Figure 3B and Supplement 14).

Three variants surpassed the genome-wide significance threshold, with rs7457539582 (p = 4.17×10^−9^) being the most significant. All three variants mapped to a 0.043 Mb QTL region on chromosome 19 (5.455–5.498 Mb), which exhibited strong LD throughout, as visualized by LD plots encompassing the lead SNP and other significant SNPs (Figure 3C, 3D). Although the genotype-phenotype association for the lead SNP did not reach statistical significance in post-hoc testing, a non-significant trend was observed: individuals homozygous for the alternative (CC) allele showed a tendency toward lower average litter sizes (Figure 3E). This QTL is located near the *TMEM100* gene. To infer potential pleiotropic functions of *TMEM100*, we performed a PheWAS using human GWAS data encompassing 190 traits across 19 domains from 45 studies. This analysis revealed significant associations of *TMEM100* with neurodevelopmental, cellular, and metabolic phenotypes (Figure 3F).

### *MAPK8* affects litter size in Leizhou goat

Integrating candidate genes from both the selective sweep and GWAS analyses revealed an overlap of only two genes: *MAPK8* and *HHAT*. Similarly, pathway enrichment analysis showed convergence, with several KEGG pathways—such as apoptosis, the dopaminergic synapse, glycosaminoglycan biosynthesis, and the MAPK signaling pathway—significantly enriched in both datasets. Notably, *MAPK8* emerged as a central component common to multiple enriched pathways. The genomic region encompassing *MAPK8* and its upstream sequence showed strong signals of positive selection, supported by significantly negative Tajima’s D values and high CLR scores (Figure 4A). Haplotype analysis revealed distinct patterns downstream of *MAPK8* between the HL and LL groups (Figure 4B). Notably, a GWAS-identified QTL for litter size precisely overlapped this selected region. The lead SNP of this QTL (rs6656908751) was in strong LD with neighboring variants (Figure 4C). We observed a significant phenotypic difference (*P* < 0.05) between the heterozygous mutant (TC) and wild-type (CC) genotypes of the lead SNP, indicating its significant role in influencing litter size in Leizhou goats (Figure 4D). An LD heatmap of the *MAPK8* locus further illustrated localized LD blocks within the gene body and its upstream regulatory region (Figure 4E). Notably, the QTL was identified within a selective sweep region upstream of *MAPK8*. The high CLR scores in this region further highlighted its functional importance. To investigate potential regulatory mechanisms, we scanned this region for conserved motifs and identified a 28-base pair candidate motif (Figure 4F). Prediction of the binding transcription factor, corroborated by database searches, indicated that this motif is a putative binding site for *GATA4*. The knockdown of *GATA4* decreases estradiol and progesterone production by suppressing the promoter activity of key steroidogenic genes [25]. To infer potential pleiotropic effects of *MAPK8*, we performed a PheWAS using a human GWAS atlas comprising 299 traits across 24 domains from 81 studies. This analysis revealed significant associations of *MAPK8* with cardiovascular, neurological, and metabolic phenotypes. Importantly, a specific association was observed with reproductive traits (p = 9.98×10^−5^) (Figure 4G). Collectively, our findings support a model wherein genetic variants in the upstream regulatory region of *MAPK8* modulate its expression, likely by altering the binding affinity of the transcription factor *GATA4*. Given that *MAPK8* encodes JNK1—a central kinase in stress response and apoptosis pathways—dysregulation of its expression may perturb germ cell homeostasis, which might ultimately be associated with litter size.

To verify whether the expression levels of the candidate genes identified from the above analyses (*BMP6*, *TMEM100*, and *MAPK8*) differed significantly between the high- and low-fertility groups in ovarian and pituitary tissues, we evaluated their differential expression. In ovarian tissues, *BMP6* expression was significantly higher in the low-fertility group compared to the high-fertility group, whereas *TMEM100* and *MAPK8* showed no significant differences. Conversely, in pituitary tissues, *BMP6* expression did not differ significantly between the two groups, whereas both *TMEM100* and *MAPK8* exhibited significant differences. Specifically, *TMEM100* expression was significantly higher in the high-fertility group, while *MAPK8* expression was significantly higher in the low-fertility group (Supplement 15). These results indicate that the candidate genes regulate goat litter size through tissue-specific expression patterns, with *BMP6* functioning primarily in the ovary and *TMEM100* alongside *MAPK8* in the pituitary.

## DISCUSSION

To unravel the genetic architecture of litter size, we performed WGS on Leizhou goats segregated into high- and low-fecundity groups. We integrated findings from selection signature analysis and GWAS to pinpoint genomic regions and candidate genes associated with litter size. Collectively, our study provides foundational genomic resources and mechanistic insights that will facilitate the genetic improvement of reproductive efficiency in goats.

Population structure analysis revealed no significant genetic differentiation between the HL and LL groups. This limited divergence likely contributed to the relatively moderate signals observed in the selective sweep analysis. However, a subtle differentiation trend was observed in the phylogenetic tree, where some branches showed an asymmetric distribution of individuals from the two groups. This pattern could signify the initial phases of artificial selection for reproductive traits, a divergence that may intensify over time. This incipient differentiation was supported by genetic diversity indices. The HL group exhibited faster LD decay, higher nucleotide diversity, and greater heterozygosity than the LL group, collectively indicating higher genetic diversity in the HL group. Furthermore, the analysis of ROH provided consistent evidence for the differing levels of inbreeding. The LL group exhibited a greater number and total length of ROHs (averaging 595.93 per individual) compared to the HL group (averaging 356.13 per individual). This pattern was reflected in the inbreeding coefficients, where the HL group showed a lower value (F_IS_ = 0.063, F_ROH_ = 0.094) than the LL group (F_IS_ = 0.138, F_ROH_ = 0.095), indicating a higher recent inbreeding pressure in the low-fertility group. Similar to our findings, ROH analysis in Yunshang black goats also revealed that the low-fertility population possesses longer ROHs and lower genetic diversity [26]. Our analysis revealed that the ROH values in the Leizhou goat population are notably higher than those reported for other breeds such as Boer [27], Saanen (107.72), and Sarda (164.65) [28], highlighting its relatively high genomic homozygosity. The F_IS_ (HL = 0.063, LL = 0.138) and F_ROH_ (HL = 0.094, LL = 0.095) values we calculated for the Leizhou goat are higher than those reported for the Ninglang Black Head goat (F_ROH_ = 0.03) and Russian local goats (F_ROH_ = 0.032–0.054), yet comparable to the Hainan black goat (F_ROH_ = 0.107). These results indicate an emerging trend of inbreeding within the Leizhou goat population, suggesting that strategies to mitigate inbreeding should be implemented in subsequent breeding programs [8,29].

By integrating analyses of *F*_ST_, θπ, and XP-EHH on genomic data from high- and low-fecundity Leizhou goats, we identified multiple selection signatures. Among these, we focused on the bone morphogenetic protein 6 (*BMP6*) gene due to its strong selection signals and enrichment in the ovarian steroidogenesis pathway. Normal ovarian function and follicular development are regulated by paracrine/autocrine factors, including bone morphogenetic proteins (BMPs). *BMP6* is a transforming growth factor beta superfamily member produced by mammalian oocytes as well as other cell types [30]. Expressed in follicular oocytes and granulosa cells (GCs), it plays roles in folliculogenesis, steroidogenesis, and follicular atresia [31]. Previous studies have demonstrated that *BMP6* is closely linked to reproductive traits in ruminant animals [32], yet the potential candidate variants remain unclear. In our study, the broad selective sweep region around *BMP6* and the observed haplotype patterns suggest that the phenotypic variation may be influenced by multiple variants with weak individual effects. We subsequently identified several SNPs within this region that showed significant allele frequency differences between the HL and LL groups, highlighting them as candidate markers for litter size in Leizhou goats. Notably, two of these SNPs (rs646562093, rs7458763602) also exhibited significant differentiation in broader validation panels of high- and low-fertility goats, underscoring their potential role in regulating litter size across breeds. However, our selection signal analysis was conducted within the Leizhou goat population, so the detected signals may be partly attributable to genetic differentiation among subpopulations rather than to population-wide selection. The candidate genes and variants still require further functional validation.

Our GWAS for litter size in Leizhou goats identified several genomic regions harboring candidate genes with uncharacterized or poorly defined roles in reproduction. The most significant association signal was mapped to a QTL on chromosome 19, located near the *TMEM100* gene. *TMEM100* encodes a transmembrane protein and plays crucial roles in the normal development of the embryonic vascular system and in pain perception [33]. Given its essential role in early embryogenesis, we hypothesize that variants with potential functional impact in *TMEM100* may compromise embryonic viability, ultimately reducing litter size. The strength of the association signal and the high LD in this region underscore its potential functional importance. Consistent with this, genotype-phenotype analysis revealed that the homozygous mutant (CC) genotype of the lead SNP was strongly associated with singleton births, suggesting a detrimental effect on litter size. Notably, the majority of GWAS signals reside in non-coding regions, highlighting the need to investigate their regulatory roles in future studies.

The low heritability of litter size complicates single-gene identification. GWAS often has limited power to detect minor-effect loci due to a low genetic signal-to-noise ratio, while selection signature analysis cannot easily distinguish direct selection on litter size from indirect selection on correlated traits. To overcome these limitations, we integrated GWAS with selection signature analysis. By identifying genes supported by both approaches, we sought convergent evidence from contemporary genetic associations and historical selection, thereby increasing confidence in pinpointing key regulators of litter size. *MAPK8* and *HHAT* were co-identified as candidate genes through both GWAS and selection signature analysis. *HHAT* encodes a palmitoyl acyltransferase that plays a critical role in embryonic development [34]. Mutations in this gene are known to cause disorders of sex development in humans, but no related studies have been conducted in animals [35]. Mitogen-activated protein kinase 8 (*MAPK8*), which encodes the kinase JNK1, is a central signal transducer. It modulates germ cell apoptosis in embryonic stem cells via the JNK pathway, and in mouse oocytes, co-activates AMPK to promote germinal vesicle breakdown [36], highlighting its conserved role in gametogenesis. Although *MAPK8* clearly influences germ cell development through multiple pathways, its precise regulatory mechanisms are not fully defined. We found that the promoter region of the *MAPK8* gene also exhibits a strong association with lambing traits and shows evidence of selection. We identified a significant motif within this region predicted to bind the transcription factor *GATA4*. *GATA4*, a member of the GATA family of zinc finger transcription factors, plays a critical role in mammalian reproductive development and function [37]. It is highly expressed in GCs but shows lower expression in the corpus luteum, indicating its important roles in folliculogenesis, luteinization, and steroidogenesis [38]. Thus, *GATA4* may contribute to *MAPK8* promoter activity and potentially activate *MAPK8* expression. Interestingly, a connection emerges with another key gene from our study, *BMP6*. Evidence suggests that *BMP6* can upregulate *GATA4* through the SMAD1/5/8 signaling pathway [39], which is crucial for ovarian function regulation [40,41]. Synthesizing these findings, we propose a hypothetical regulatory model: *BMP6* signals through SMAD1/5/8 to upregulate *GATA4*, which then may transcriptionally activate *MAPK8* expression. This *MAPK8* activation could, in turn, affect germ cell development and survival, which might be associated with litter size.

## CONCLUSION

In conclusion, this study, whole-genome resequencing of 92 Leizhou goats—comprising 30 high- and 62 LL individuals—yielded 20.8 million high-quality SNPs, enabling detailed analysis of population structure. The elevated genetic diversity in the HL group underscores its potential for future selective breeding and highlights its value for genetic conservation. Integrating selection signature analysis and GWAS, we identified *BMP6*, *TMEM100*, and *MAPK8* as candidate genes and further suggested a potential role of the *BMP6*-*GATA4*-*MAPK8* signaling pathway in goat litter size. Furthermore, the genetic variants and candidate genes identified in this study provide a foundation for elucidating the molecular mechanisms underlying reproductive traits and facilitate molecular breeding programs in goats.

## Figures and Tables

**Figure 1:**
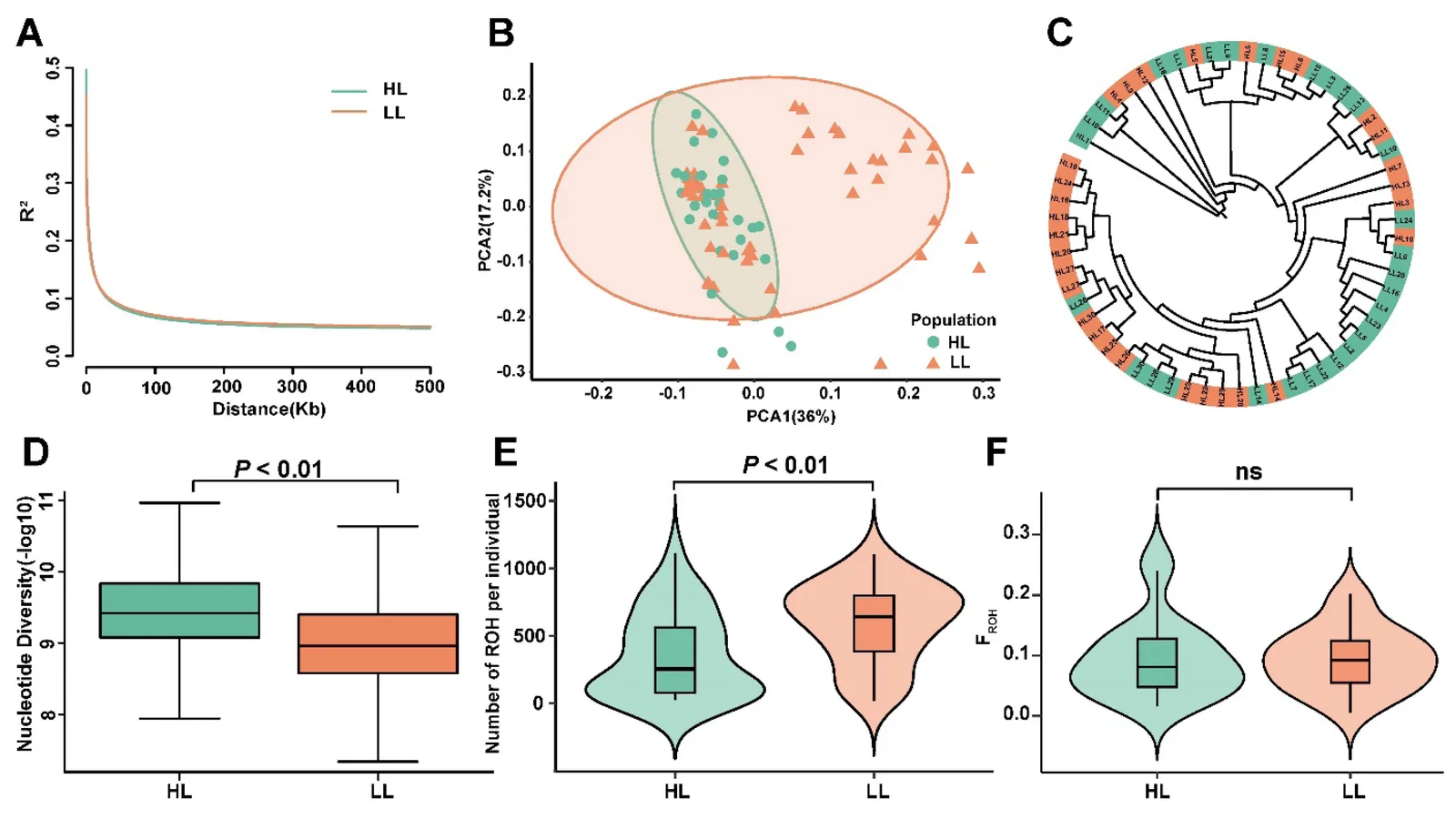
Genetic diversity and population structure analysis for leizhou goat populations. (A) Extent of linkage disequilibrium (LD) in high and low litter size groups of Leizhou goats. Values are the mean LD r^2^ values for all pairs of SNPs binned by distance. (B) Principal component analysis (PCA). (C) Neighbor-joining tree of Leizhou goat. (D) Nucleotide diversity (20 kb windows). (E) The inbreeding coefficient calculated based on runs of homozygosity (ROH). (F) ROH distribution across the genome for the high-litter-size Leizhou goat populations. ROH distribution for each chromosome considering different ROH length classes for different goat breeds. HL, high litter size; LL, low litter size; SNP, single nucleotide polymorphism.

**Figure 2:**
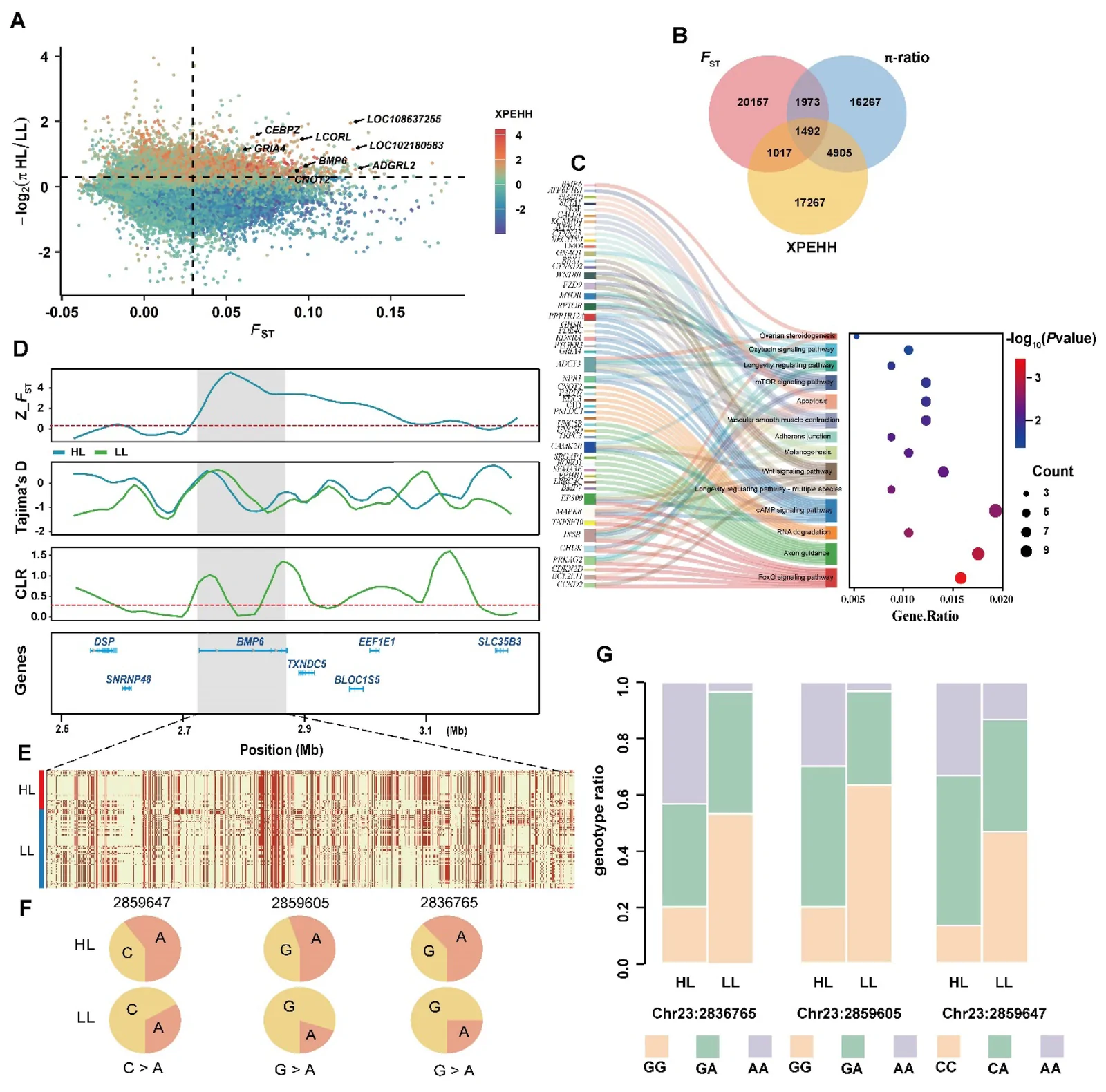
Positive selection signature for high and low litter size Leizhou goat populations. (A) Distribution of the pairwise fixation index (*F*_ST_) (x axis), log2π-ratio (y axis) and value of XP-EHH (color) between high and low litter size Leizhou goats. The dashed vertical and horizontal lines indicate the significance threshold (*F*_ST_ = 0.03, log_2π_-ratio = 0.3, XP-EHH = 1.41) used for extracting outliers. (B) Venn diagrams. Numbers in the intersection regions overlap regions among the selection regions based on pairwise fixation index (*F*_ST_), log_2π_-ratio, and XP-EHH. (C) Major enriched KEGG pathways of identified selected genes. (D) Selection signature and distribution on chromosome 23 (2.5 to 3.2 Mb). The putative sweep region is additionally validated by *Z*_*F*_ST_, Tajimas D, and CLR test. Gene annotations in the sweep region are indicated at the bottom. (E) SNPs with minor allele frequencies >0.05 were used to construct haplotype patterns for *BMP6* (Chr 23:2.72–2.87 Mb). (F) Allele frequencies of 3 SNPs in the *BMP6* gene in high and low litter size Leizhou goat populations. The red alleles are consistent with reference genome, and the blue alleles represent alternative alleles. (G) Genotype of 3 SNPs in the *BMP6* gene in high and low litter size Leizhou goat populations. HL, high litter size; LL, low litter size; KEGG, Kyoto Encyclopedia of Genes and Genomes; CLR, Composite Likelihood Ratio; SNP, single nucleotide polymorphism.

**Figure 3:**
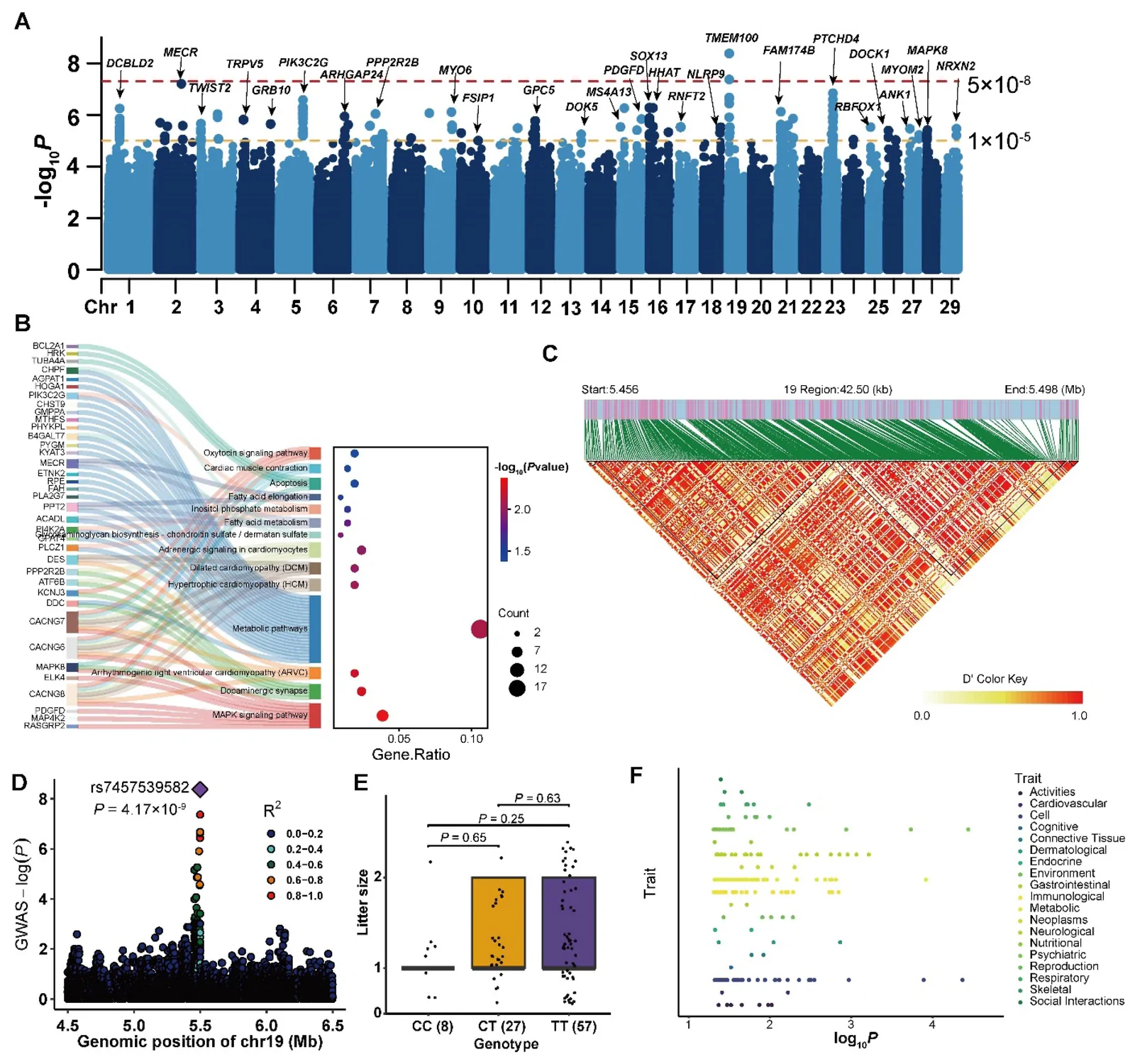
Genome wide association study (GWAS) and QTL candidate region annotation. (A) Manhattan plot of association signals for the litter size phenotype based on whole-genome SNPs. The horizontal dashed lines correspond to the genome-wide significance threshold 5×10^−8^ and 1×10^−5^ for SNPs. (B) Major enriched KEGG pathways of identified candidate genes. (C) Heatmap showing the LD in the most significant QTL region identified by GWAS. Darker colors indicate higher degrees of LD. (D) The extent of LD between the lead SNP (rs7457539582) of the most significant QTL from the GWAS and the surrounding SNPs. (E) The boxplot shows the association between lead SNP (rs7457539582) genotypes and litter size. (F) Manhattan plot from the phenome-wide association analysis of the *TMEM100* gene. QTL, quantitative trait locus; SNP, single nucleotide polymorphism; KEGG, Kyoto Encyclopedia of Genes and Genomes; LD, linkage disequilibrium.

**Figure 4:**
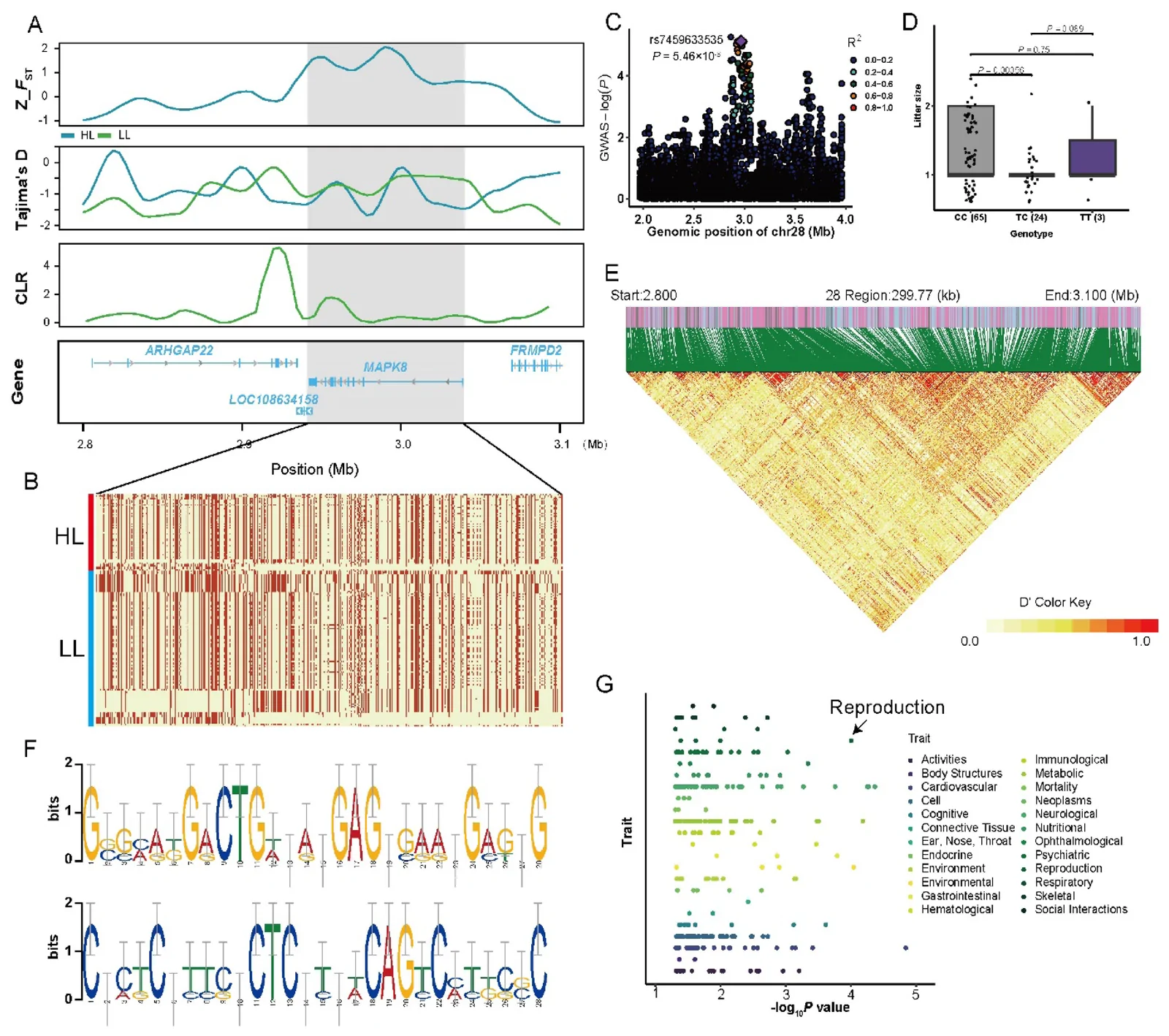
The association between *MAPK8* and litter size trait in goats. (A) Selection signature and distribution on chromosome 28 (2.8 to 3.1 Mb). The putative sweep region is additionally validated by *Z*_*F*_ST_, Tajimas D, and CLR test. Gene annotations in the sweep region are indicated at the bottom. (B) SNPs with minor allele frequencies >0.05 were used to construct haplotype patterns for *MAPK8* (Chr 28:2.8–3.1 Mb). (C) The extent of LD between the lead SNP (rs7459633535) of the significant QTL from the GWAS and the surrounding SNPs. (D) The boxplot shows the association between lead SNP (rs7459633535) genotypes and litter size. (E) Heatmap shows the degree of LD in the region of *MAPK8* gene. Darker colors indicate higher degrees of LD. (F) Significant motifs identified in the *MAPK8* gene promoter region. (G) Manhattan plot from the phenome-wide association analysis of the *MAPK8* gene. CLR, Composite Likelihood Ratio; GWAS, genome-wide association study; HL, high litter size; LL, low litter size; SNP, single nucleotide polymorphism; LD, linkage disequilibrium; QTL, quantitative trait locus.

**Table 1:** Descriptive statistics of genetic diversity indicators in the HL and LL groups of Leizhou goats

Population	H_O_	H_E_	PIC	A	Ae	F_IS_	MAF	HWE
HL	0.177	0.176	0.150	1.454	1.120	0.063	0.054	0.945
LL	0.155	0.155	0.132	1.509	1.118	0.138	0.053	0.933

H_O_, observed heterozygosity; H_E_, expected heterozygosity; PIC, polymorphism information content; A, observed number of alleles; Ae, effective number of alleles; F_IS_, inbreeding coefficient based on heterozygosity; MAF, minor allele frequency; HWE, Hardy-Weinberg Equilibrium; HL, high litter size; LL, low litter size.

## Data Availability

The raw sequence data reported in this paper have been deposited in the Genome Sequence Archive in National Genomics Data Center, China National Center for Bioinformation/Beijing Institute of Genomics, Chinese Academy of Sciences (GSA: CRA039898) that are publicly accessible at https://ngdc.cncb.ac.cn/gsa .

## SUPPLEMENTARY MATERIAL

**Supplement 1**. A phenotypic dataset of litter size for 92 Leizhou goats.

**Supplement 2**. Distribution of SNPs identified in Leizhou goats within various genomic regions annotated by ANNOVAR.

**Supplement 3**. The distribution of SNPs in Leizhou goat chromosomes and their genomic region annotation.

**Supplement 4**. Distribution of SNPs identified in Leizhou goats within various exonic regions annotated by ANNOVAR.

**Supplement 5**. Autosomal regions identified as candidate selection sweeps by comparing genetic variants between 30 high litter size and 30 low litter size in Leizhou goats.

**Supplement 6**. Results of KEGG enrichment analysis for the 346 candidate genes identified by selection signature analysis.

**Supplement 7**. Selective sweep analysis by comparing genomes between high and low litter size Leizhou goats.

**Supplement 8**. Selective sweep analysis by comparing genomes between high (n=30) and low (n=62) litter size Leizhou goats.

**Supplement 9**. The distribution of genotypes for the three key SNPs (A. rs7458763602. B. rs646562093. C. rs7458736596.) in the *BMP6* gene across high and low reproductive goat populations.

**Supplement 10**. Quantile-quantile (Q-Q) plot of association signals for the litter size phenotype based on GEMMA.

**Supplement 11**. Manhattan plot of association signals for the litter size phenotype based on whole-genome SNPs.

**Supplement 12**. Quantile-quantile (Q-Q) plot of association signals for the litter size phenotype based on Plink2.

**Supplement 13**. A GWAS on litter size traits in 92 Leizhou goats identified significant autosomal QTL.

**Supplement 14**. Results of KEGG enrichment analysis for the 207 candidate genes identified by GWAS.

**Supplement 15**. Differential expression of *BMP6, TMEM100*, and *MAPK8* genes in ovarian and pituitary tissues.
