# Supplementary material for: Integrating selective signature analysis and genome-wide association study to identify candidate genes associated with litter size in Leizhou goat
Source: Anim Biosci. 2026 Jul 27;39(11):260280. doi: 10.5713/ab.260280 (PMC13740650; doi:10.5713/ab.260280)
Supplement: Supplementary file 1 [file ab-260280-Supplementary-1.pdf]

Supplementary Material

Supplement 1. A phenotypic dataset of litter size for 92 Leizhou goats.

Individual	First parity	Second parity	Third parity	Average litter size	Farm
LZ1	1	1	1	1	Farmers in Dongfang City, Hainan Province
LZ2	2	2	3	2.33	Farmers in Dongfang City, Hainan Province
LZ3	1	1	1	1	Farmers in Dongfang City, Hainan Province
LZ4	2	2	3	2.33	Zhanjiang Leizhou Goat Breeding Farm Guangdong Ocean University Branch
LZ5	1	3	3	2.33	Zhanjiang Leizhou Goat Breeding Farm Guangdong Ocean University Branch
LZ6	1	1	1	1	Farmers in Dongfang City, Hainan Province
LZ7	1	1	1	1	Farmers in Dongfang City, Hainan Province
LZ8	1	1	1	1	Farmers in Dongfang City, Hainan Province
LZ9	2	2	2	2	Farmers in Dongfang City, Hainan Province
LZ10	2	2	2	2	Farmers in Dongfang City, Hainan Province
LZ11	2	2	2	2	Farmers in Leizhou City 1
LZ12	2	3	3	2.67	Farmers in Leizhou City 1
LZ13	2	3	2	2.33	Farmers in Leizhou City 1
LZ14	1	2	3	2	Farmers in Leizhou City 2
LZ15	1	2	3	2	Farmers in Leizhou City 1
LZ16	2	2	2	2	Farmers in Leizhou City 1
LZ17	1	2	3	2	Farmers in Leizhou City 1
LZ18	2	2	2	2	Farmers in Leizhou City 1
LZ19	2	3	2	2.33	Farmers in Leizhou City 1
LZ20	2	2	2	2	Farmers in Leizhou City 2
LZ21	2	2	3	2.33	Farmers in Leizhou City 2
LZ22	1	3	3	2.33	Farmers in Leizhou City 2
LZ23	2	2	2	2	Farmers in Leizhou City 2
LZ24	1	1	1	1	Farmers in Leizhou City 2
LZ25	1	1	1	1	Farmers in Leizhou City 2
LZ26	2	2	2	2	Farmers in Leizhou City 2
LZ27	1	3	3	2.33	Farmers in Leizhou City 2
LZ28	2	2	3	2.33	Farmers in Leizhou City 2
LZ29	1	2	3	2	Farmers in Dongfang City, Hainan Province
LZ30	1	1	1	1	Farmers in Dongfang City, Hainan Province
LZ31	2	3	2	2.33	Farmers in Dongfang City, Hainan Province
LZ32	2	2	2	2	Farmers in Dongfang City, Hainan Province
LZ33	1	1	1	1	Farmers in Dongfang City, Hainan Province
LZ34	1	1	1	1	Farmers in Dongfang City, Hainan Province
LZ35	1	1	1	1	Farmers in Dongfang City, Hainan Province

LZ36	1	1	1	1	Farmers in Dongfang City, Hainan Province
LZ37	1	1	1	1	Farmers in Dongfang City, Hainan Province
LZ38	1	1	1	1	Farmers in Dongfang City, Hainan Province
LZ39	1	1	1	1	Farmers in Dongfang City, Hainan Province
LZ40	1	1	1	1	Zhanjiang Leizhou Goat Breeding Farm Guangdong Ocean University Branch
LZ41	1	1	1	1	Zhanjiang Leizhou Goat Breeding Farm Guangdong Ocean University Branch
LZ42	1	1	1	1	Zhanjiang Leizhou Goat Breeding Farm Guangdong Ocean University Branch
LZ43	1	1	1	1	Zhanjiang Leizhou Goat Breeding Farm Guangdong Ocean University Branch
LZ44	1	1	1	1	Zhanjiang Leizhou Goat Breeding Farm Guangdong Ocean University Branch
LZ45	1	3	3	2.33	Jiaoyiling Black Goat Breeding Farm in Zhanjiang City
LZ46	2	2	3	2.33	Jiaoyiling Black Goat Breeding Farm in Zhanjiang City
LZ47	2	3	3	2.67	Jiaoyiling Black Goat Breeding Farm in Zhanjiang City
LZ48	1	3	3	2.33	Jiaoyiling Black Goat Breeding Farm in Zhanjiang City
LZ49	2	2	3	2.33	Jiaoyiling Black Goat Breeding Farm in Zhanjiang City
LZ50	2	2	3	2.33	Jiaoyiling Black Goat Breeding Farm in Zhanjiang City
LZ51	1	1	1	1	Jiaoyiling Black Goat Breeding Farm in Zhanjiang City
LZ52	1	1	1	1	Jiaoyiling Black Goat Breeding Farm in Zhanjiang City
LZ53	1	1	1	1	Jiaoyiling Black Goat Breeding Farm in Zhanjiang City
LZ54	1	1	1	1	Jiaoyiling Black Goat Breeding Farm in Zhanjiang City
LZ55	1	1	1	1	Jiaoyiling Black Goat Breeding Farm in Zhanjiang City
LZ56	1	1	1	1	Jiaoyiling Black Goat Breeding Farm in Zhanjiang City
LZ57	1	1	1	1	Jiaoyiling Black Goat Breeding Farm in Zhanjiang City
LZ58	1	1	1	1	Jiaoyiling Black Goat Breeding Farm in Zhanjiang City
LZ59	1	1	1	1	Jiaoyiling Black Goat Breeding Farm in Zhanjiang City
LZ60	1	1	1	1	Jiaoyiling Black Goat Breeding Farm in Zhanjiang City
LZ61	1	1	1	1	Jiaoyiling Black Goat Breeding Farm in Zhanjiang City
LZ62	1	1	1	1	Jiaoyiling Black Goat Breeding Farm in Zhanjiang City
LZ63	1	1	1	1	Jiaoyiling Black Goat Breeding Farm in Zhanjiang City
LZ64	1	1	1	1	Jiaoyiling Black Goat Breeding Farm in Zhanjiang City
LZ65	1	1	1	1	Jiaoyiling Black Goat Breeding Farm in Zhanjiang City
LZ66	1	1	1	1	Jiaoyiling Black Goat Breeding Farm in Zhanjiang City
LZ67	1	1	1	1	Jiaoyiling Black Goat Breeding Farm in Zhanjiang City
LZ68	1	1	1	1	Jiaoyiling Black Goat Breeding Farm in Zhanjiang City
LZ69	1	1	1	1	Jiaoyiling Black Goat Breeding Farm in Zhanjiang City
LZ70	1	1	1	1	Jiaoyiling Black Goat Breeding Farm in Zhanjiang City
LZ71	1	1	1	1	Zhanjiang Experimental Station of Chinese Academy of Tropical Sciences
LZ72	1	1	1	1	Zhanjiang Experimental Station of Chinese Academy of Tropical Sciences

LZ73	1	1	1	1	Zhanjiang Experimental Station of Chinese Academy of Tropical Sciences
LZ74	1	1	1	1	Zhanjiang Experimental Station of Chinese Academy of Tropical Sciences
LZ75	1	1	1	1	Zhanjiang Experimental Station of Chinese Academy of Tropical Sciences
LZ76	1	1	1	1	Zhanjiang Experimental Station of Chinese Academy of Tropical Sciences
LZ77	1	1	1	1	Zhanjiang Experimental Station of Chinese Academy of Tropical Sciences
LZ78	1	1	1	1	Zhanjiang Experimental Station of Chinese Academy of Tropical Sciences
LZ79	1	1	1	1	Zhanjiang Experimental Station of Chinese Academy of Tropical Sciences
LZ80	1	1	1	1	Zhanjiang Experimental Station of Chinese Academy of Tropical Sciences
LZ81	1	1	1	1	Zhanjiang Experimental Station of Chinese Academy of Tropical Sciences
LZ82	1	1	1	1	Zhanjiang Experimental Station of Chinese Academy of Tropical Sciences
LZ83	1	1	1	1	Zhanjiang Experimental Station of Chinese Academy of Tropical Sciences
LZ84	1	1	1	1	Zhanjiang Experimental Station of Chinese Academy of Tropical Sciences
LZ85	1	1	1	1	Zhanjiang Experimental Station of Chinese Academy of Tropical Sciences
LZ86	1	1	1	1	Zhanjiang Experimental Station of Chinese Academy of Tropical Sciences
LZ87	1	1	1	1	Zhanjiang Experimental Station of Chinese Academy of Tropical Sciences
LZ88	1	1	1	1	Zhanjiang Experimental Station of Chinese Academy of Tropical Sciences
LZ89	1	1	1	1	Zhanjiang Experimental Station of Chinese Academy of Tropical Sciences
LZ90	1	1	1	1	Zhanjiang Experimental Station of Chinese Academy of Tropical Sciences
LZ91	1	1	1	1	Zhanjiang Experimental Station of Chinese Academy of Tropical Sciences
LZ92	1	1	1	1	Zhanjiang Experimental Station of Chinese Academy of Tropical Sciences
