# Supplementary material for: Integrating selective signature analysis and genome-wide association study to identify candidate genes associated with litter size in Leizhou goat
Source: Anim Biosci. 2026 Jul 27;39(11):260280. doi: 10.5713/ab.260280 (PMC13740650; doi:10.5713/ab.260280)
Supplement: Supplementary file 3 [file ab-260280-Supplementary-11.pdf]

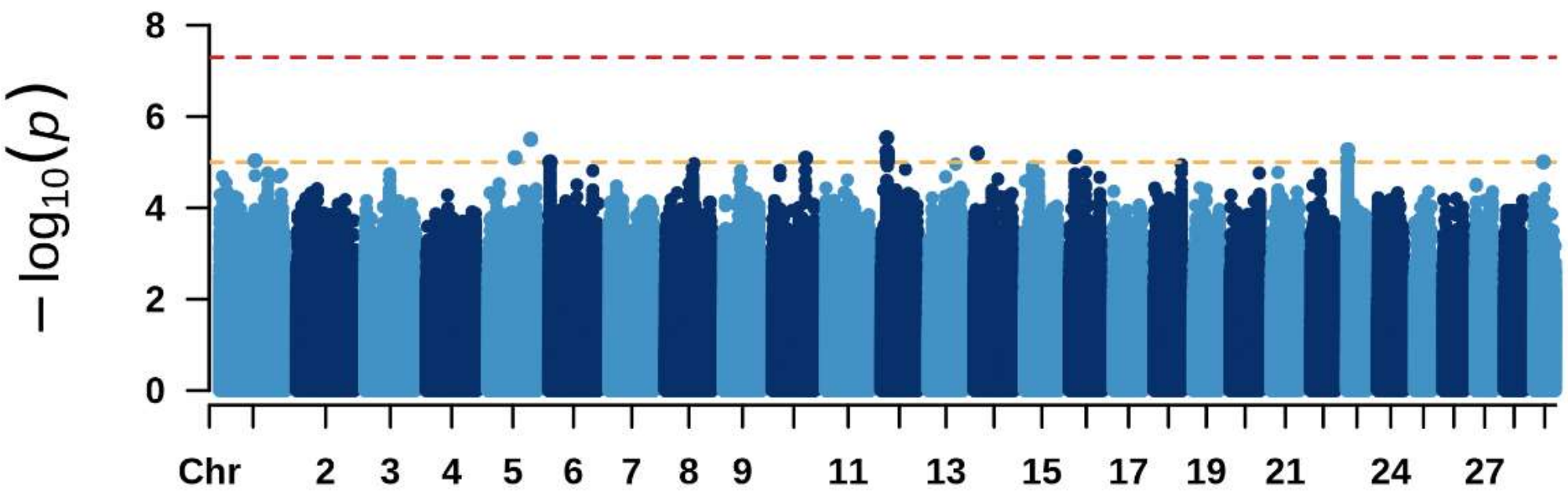

Supplement 11. Manhattan plot of association signals for the litter siz e phenotype based on whole-genome SNPs. The horizontal dashed lines correspond to the genome-wide significance threshold 5×10^{-8} and 1×10^{-5} for SNPs.
