# Supplementary material for: Integrating selective signature analysis and genome-wide association study to identify candidate genes associated with litter size in Leizhou goat
Source: Anim Biosci. 2026 Jul 27;39(11):260280. doi: 10.5713/ab.260280 (PMC13740650; doi:10.5713/ab.260280)
Supplement: Supplementary file 4 [file ab-260280-Supplementary-12.pdf]

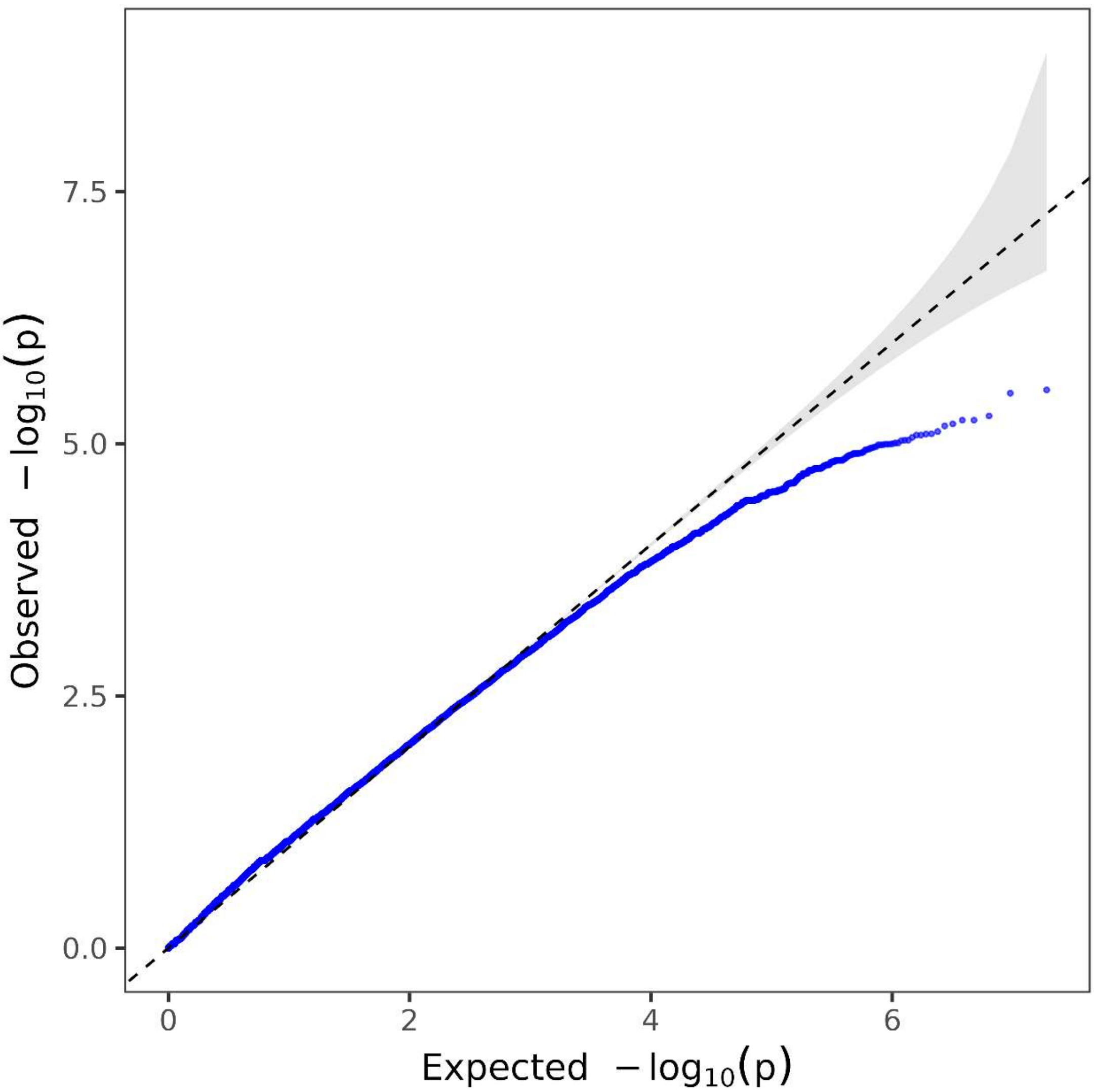

Supplement 12. Quantile-quantile (Q-Q) plot of association signals for the litter size phenotype based on Plink2.
