# Supplementary material for: Integrating selective signature analysis and genome-wide association study to identify candidate genes associated with litter size in Leizhou goat
Source: Anim Biosci. 2026 Jul 27;39(11):260280. doi: 10.5713/ab.260280 (PMC13740650; doi:10.5713/ab.260280)
Supplement: Supplementary file 5 [file ab-260280-Supplementary-13.pdf]

Supplement 13. A GWAS on litter size traits in 92 Leizhou goats identified significant autosomal QTL. For each QTL, the lead SNP was determined, along with the genomic start and end positions of all QTL regions. The functions of related genes were then annotated through literature search.

NO	Chr	Lead snp	QTLL	QTLR	*P*	Protein-coding gene	Related Phenotype
1	1	43,112,701	43,091,018	43,132,361	5.64E-07	*DCBLD2,COL8A1*	1.External pelvimetry of cattle[1]<br>2.Total lactation milk yield[2]
2	2	90,172,210	90,172,210	90,172,210	6.23E-08	*RND3*	-
3	2	11,635,609	11,635,609	11,635,609	2.02E-06	*MECR*	Residual feed intake[3]
4	2	95,061,980	95,010,822	95,061,980	2.63E-06	*KCNJ3*	Temperament of cattle[4]
5	2	37,895,688	37,895,688	37,895,688	2.76E-06	*ACADL,KANSL1L*	Intramuscular fat[5]
6	2	28,517,920	28,482,486	28,517,920	6.71E-06	*DNPEP*	-
7	3	63,261,031	63,261,031	63,261,568	9.31E-07	*COL24A1*	Osteochondrosis of horse[6]
8	3	1,334,461	1,334,166	1,343,542	2.2E-06	-	-
9	3	66,452,696	66,452,696	66,452,700	8.58E-06	*GBP5*	-
10	3	2,686,150	2,686,150	2,686,150	9.33E-06	*TWIST2,LOC102179832*	-
11	4	14,381,412	14,381,412	14,384,554	1.53E-06	*LLCFC1,TRPV5*	Ca metabolism of cattle[7]
12	4	115,202,147	115,202,147	115,204,169	2.25E-06	*DDC,GRB10*	1.Skeletal muscle growth of pig[8]

							2.Birth weight of sheep[9] 3.Reproductive of pig[10]
13	5	90,096,298	90,025,708	90,102,612	2.64E-07	*PIK3C2G*	1.Altitude adaptation of sheep[11] 2.Heat stress response of saturated fatty acids[12]
14	6	100,969,770	100,969,770	100,969,770	1.15E-06	*ARHGAP24,MAPK10,LOC102189022*	Growth of sheep[13]
15	6	114,309,995	114,309,995	114,309,995	2.41E-06	*SORCS2,AFAP1,ABLIM2*	-
16	7	73,345,791	73,345,791	73,357,583	9.12E-07	-	-
17	7	52,527,731	52,527,731	52,537,567	2.66E-06	*PPP2R2B,GPR151,TCERG1*	Gestation length of pig[14]
18	7	99,246,812	98,746,812	99,250,055	5.37E-06	*ZNF317*	-
19	7	80,002,232	80,002,232	80,010,718	8.91E-06	*PRDM6*	Body size of goat[15]
20	7	71,495,941	71,495,941	71,495,941	9.15E-06	*N4BP3,B4GALT7,TMED9*	Leg length of goat[16]
21	8	72,783,714	72,783,714	72,783,714	7.85E-06	-	-
22	9	84,671,873	84,671,873	84,671,873	7.76E-07	-	-
23	9	2,672,598	2,672,598	2,672,598	8.55E-07	*MYO6,SENP6,IMPG1*	Wool color of sheep[17]
24	9	86,774,874	86,774,874	86,782,385	2.55E-06	-	-
25	10	4,703,343	4,703,343	4,703,343	5.01E-06	-	-
26	10	67,805,821	67,805,821	67,805,821	9.9E-06	*FSIP1,LOC102174478*	-
27	11	88,858,438	88,858,438	88,858,540	3.26E-06	-	-
28	12	22,539,306	22,539,306	22,573,760	1.71E-06	-	-

29	12	14,891,626	14,891,626	14,891,666	5.27E-06	-	-
30	12	19,700,838	19,700,838	19,700,838	8.36E-06	*GPC5*	Nipple number of sheep[18]
31	13	81,560,708	81,560,708	81,564,763	5.66E-06	*DOK5*	Body size of cattle[19]
32	15	14,475,721	14,475,721	14,475,721	5.46E-07	-	-
33	15	78,249,856	78,249,856	78,249,856	1.46E-06	*PDGFD*	1.Fat tail of sheep[20] 2.Litter size of sheep[21]
34	15	193,329	193,329	193,329	2.94E-06	*MS4A13,MS4A1*	-
35	15	61,243,057	61,243,057	61,243,057	3.91E-06	*POU2AF2*	-
36	16	1,229,650	1,214,065	1,236,675	5.28E-07	*SOX13*	Fat deposition of pig[22]
37	16	14,356,875	14,356,875	14,363,570	5.33E-07	*BRINP3*	1.Services per conception of cattle[23] 2.Total teat number of pig[24]
38	16	71,525,569	71,525,569	71,525,569	4.68E-06	*HHAT*	-
39	16	19,063,048	19,063,048	19,063,083	6.48E-06	-	-
40	16	2,771,841	2,771,841	2,771,841	6.92E-06	*MFSD4A,ELK4*	Milk production[25]
41	17	12,956,627	12,956,627	12,994,088	2.97E-06	*SPRING1,RNFT2*	
42	18	65,762,467	65,762,467	65,762,671	3.03E-06	-	-
43	18	63,547,369	63,547,369	63,547,378	4.92E-06	*NLRP9,RFPL4A,EPN1*	Litter size of pig[26]
44	19	5,498,066	5,455,751	5,498,253	4.17E-09	*TMEM100*	1.Embryonic development[27] 2.Pain hypersensitivity[28]

45	19	7,869,751	7,869,751	7,877,341	2.99E-06	*MSI2*	
46	21	13,576,742	13,550,155	13,576,775	7.53E-07	*FAM174B*	Meat quality of cattle[29]
47	21	58,824,410	58,709,315	58,824,410	1.39E-06	-	-
48	21	39,984,862	39,984,862	40,092,239	2.53E-06	-	-
49	21	25,618,701	25,618,701	25,618,701	3.32E-06	*BCL2A1,ZFAND6*	Trophectoderm of pig[30]
50	21	22,798,551	22,798,551	22,798,551	8.75E-06	-	-
51	23	27,751,064	27,677,432	28,038,312	1.44E-07	*PTCHD4,LOC108633562*	-
52	23	29,105,676	29,105,676	29,105,676	1.03E-06	*CYP39A1,RCAN2*	
53	23	22,811,512	22,811,512	22,811,512	5.54E-06	-	-
54	24	30,281,570	30,281,570	30,281,570	9.14E-06	*AQP4,CHST9*	-
55	25	6,629,358	6,629,358	6,629,358	2.97E-06	*RBFOX1*	-
56	26	5,460,255	5,460,255	5,467,876	4.07E-06	*DOCK1,INSYN2A*	Intramuscular fat of pig[31]
57	26	32,750,919	32,750,919	32,756,568	9.52E-06	*MARVELD1,AVPI1*	Daughter pregnancy rate of cattle[32]
58	27	8,998,840	8,998,840	8,998,840	3.43E-06	*ANK1*	Fat of cattle[33]
59	27	43,398,881	42,899,599	43,398,881	5.97E-06	*ARHGEF10,MYOM2,KBTBD11,CLN8*	Skeletal muscle development of pig[34]
60	28	4,749,419	4,749,419	4,749,419	3.87E-06	*ARHGAP22,MAPK8*	1.Reproductive of pig[35] 2.Reproductive of sheep[36] 3.Reproductive of goat[37]

							4.Heat tolerance of goat[38]
61	28	2,864,659	2,864,659	2,993,116	5.46E-06	-	-
62	29	43,295,532	43,257,947	43,295,532	3.42E-06	*SLC22A12,NRXN2*	Hoof health of cattle[39]

Reference

1.Spătaru II, Mizeranschi AE, Ilie DE, et al. Study of the Association Between SNPs and External Pelvimetry Measurements in Romanian Simmental Cattle. Animals (Basel). 2025;15(11):1586. Published 2025 May 29. doi:10.3390/ani15111586

2.Ahmad SF, Singh A, Gangwar M, Kumar S, Dutt T, Kumar A. Haplotype-based association study of production and reproduction traits in multigenerational Vrindavani population. Gene. 2023;867:147365. doi:10.1016/j.gene.2023.147365"

3.Serna-García M, Fonseca LFS, Panadero Romero JJ, et al. Transcriptome Profiling of the Liver in Nellore Cattle Phenotypically Divergent for RFI in Two Genetic Groups. Animals (Basel). 2023;13(3):359. Published 2023 Jan 20. doi:10.3390/ani13030359

4.Chen SY, Oliveira HR, Schenkel FS, Pedrosa VB, Melka MG, Brito LF. Using imputed whole-genome sequence variants to uncover candidate mutations and genes affecting milking speed and temperament in Holstein cattle. J Dairy Sci. 2020;103(11):10383-10398. doi:10.3168/jds.2020-18897

5.Wang S, Liu T, Peng P, et al. Integrated Transcriptomic Analysis of Liver and Muscle Tissues Reveals Candidate Genes and Pathways Regulating Intramuscular Fat Deposition in Beef Cattle. Animals (Basel). 2025;15(9):1306. Published 2025 Apr 30. doi:10.3390/ani15091306

6.Martinez-Saez L, Marín-García PJ, Llobat ML. Osteochondrosis in horses: An overview of genetic and other factors. Equine Vet J. 2025 Apr 29. doi: 10.1111/evj.14518.

7.Vieira-Neto A, Poindexter MB, Nehme Marinho M, Zimpel R, Husnain A, Silva ACM, Prim JG, Nelson CD, Santos JEP. Effect of source and amount of vitamin D on function and mRNA expression in immune cells in dairy cows. J Dairy Sci. 2021 Oct;104(10):10796-10811. doi: 10.3168/jds.2021-20284.

8.Li M, Liu H, Lu T, et al. Insights into tissue- and cell type-specific effects of Grb10 on pig skeletal muscle growth by multi-omics analysis. Sci China Life Sci. 2025;68(9):2659-2675. doi:10.1007/s11427-024-2910-7

9.Ghasemi M, Zamani P, Vatankhah M, Abdoli R. Genome-wide association study of birth weight in sheep. Animal. 2019 Sep;13(9):1797-1803. doi: 10.1017/S1751731118003610.

10.Wang Y, Ding X, Tan Z, Xing K, Yang T, Wang Y, Sun D, Wang C. Genome-wide association study for reproductive traits in a Large White pig population. Anim Genet. 2018 Apr;49(2):127-131. doi: 10.1111/age.12638."

11.Song Y, Yuan C, Guo T, et al. Genome-wide selection signal analysis reveals the adaptability of Tibetan sheep to high altitudes. Front Vet Sci. 2025;12:1632017. Published 2025 Aug 14. doi:10.3389/fvets.2025.1632017

12.Bohlouli M, Halli K, Yin T, Gengler N, König S. Genome-wide associations for heat stress response suggest potential candidate genes underlying milk fatty acid composition in dairy cattle. J Dairy Sci. 2022 Apr;105(4):3323-3340. doi: 10.3168/jds.2021-21152. "
13.Shan H, Guo LY, Huang X, et al. Novel polymorphism at ARHGAP24 gene and its association with growth traits in Hu sheep. Anim Biotechnol. 2025;36(1):2513958. doi:10.1080/10495398.2025.2513958
14.Wang X, Wang L, Shi L, et al. GWAS of Reproductive Traits in Large White Pigs on Chip and Imputed Whole-Genome Sequencing Data. Int J Mol Sci. 2022;23(21):13338. Published 2022 Nov 1. doi:10.3390/ijms232113338
15.Manjutha S, Elayadeth-Meethal M, Liz Abraham B, Asaf M, Senthil Murugan S, Radhika G. Screening of InDel variants in PRDM6, myostatin and IGF2BP1 genes and association analysis with body measurement traits in Malabari and Attappadi black goats. Anim Biotechnol. 2023;34(9):4760-4774. doi:10.1080/10495398.2023.2189916
16.Liu J, Dong S, Lv J, et al. Screening of SNP Loci Related to Leg Length Trait in Leizhou Goats Based on Whole-Genome Resequencing. Int J Mol Sci. 2024;25(22):12450. Published 2024 Nov 20. doi:10.3390/ijms252212450
17.Zhang W, Luosang C, Yuan C, et al. Selection signatures of wool color in Gangba sheep revealed by genome-wide SNP discovery. BMC Genomics. 2024;25(1):606. Published 2024 Jun 17. doi:10.1186/s12864-024-10464-2
18.Xie S, Isaacs K, Becker G, Murdoch BM. A computational framework for improving genetic variants identification from 5,061 sheep sequencing data. J Anim Sci Biotechnol. 2023;14(1):127. Published 2023 Oct 2. doi:10.1186/s40104-023-00923-3
19.Wang D, Zhang T, Zhang M, et al. Identification of Genomic Structural Variations in Xinjiang Brown Cattle by Deep Sequencing and Their Association with Body Conformation Traits. Int J Mol Sci. 2025;26(11):5234. Published 2025 May 29. doi:10.3390/ijms26115234
20.Jin M, Liu G, Liu E, et al. Genomic insights into the population history of fat-tailed sheep and identification of two mutations that contribute to fat tail adipogenesis. J Adv Res. Published online May 6, 2025. doi:10.1016/j.jare.2025.05.011
21.Su P, Luo Y, Huang Y, et al. Short variation of the sheep PDGFD gene is correlated with litter size. Gene. 2022;844:146797. doi:10.1016/j.gene.2022.146797"
22.Chen W, Yang F, Liufu S, Li Z, Gong Y, Ma H. Integrated analysis of muscle lncRNA and mRNA of Chinese indigenous breed Ningxiang pig in four developmental stages. Front Vet Sci. 2024;11:1465389. Published 2024 Oct 21. doi:10.3389/fvets.2024.1465389
23.Lee SH, Lee D, Lee M, Ryoo SH, Seo S, Choi I. Analysis of single nucleotide polymorphisms related to heifer fertility in Hanwoo (Korean cattle). Anim Biotechnol. 2022;33(5):964-969. doi:10.1080/10495398.2020.1856124
24.Qiu Z, Yin Y, Huang R, et al. Multiple association studies identify 3 novel candidate genes for teat number trait in Danish Landrace and Large White pigs: BRINP3, LIN52, and UBE3B. J Anim Sci. 2025;103:skaf145. doi:10.1093/jas/skaf145"
25.Iung LHS, Petrini J, Ramírez-Díaz J, et al. Genome-wide association study for milk production traits in a Brazilian Holstein population. J Dairy Sci. 2019;102(6):5305-5314.

doi:10.3168/jds.2018-14811
26.Zhang, Y., Song, Y., Zhang, W., **ao, T., & Peng, H. (2023). Effect of NLR family pyrin domain containing 9 gene polymorphism on litter size in large white pigs. Animal Biotechnology, 34(9), 4547-4552.
27.Somekawa S, Imagawa K, Hayashi H, et al. Tmem100, an ALK1 receptor signaling-dependent gene essential for arterial endothelium differentiation and vascular morphogenesis. Proc Natl Acad Sci U S A. 2012;109(30):12064-12069. doi:10.1073/pnas.1207210109
28.Nees TA, Wang N, Adamek P, et al. Role of TMEM100 in mechanically insensitive nociceptor un-silencing. Nat Commun. 2023;14(1):1899. Published 2023 Apr 5. doi:10.1038/s41467-023-37602-w"
29.Naserkheil M, Mehrban H, Lee D, Park MN. Genome-wide Association Study for Carcass Primal Cut Yields Using Single-step Bayesian Approach in Hanwoo Cattle. Front Genet. 2021 Nov 26;12:752424. doi: 10.3389/fgene.2021.752424.
30.Bou G, Guo J, Liu S, et al. OCT4 expression transactivated by GATA protein is essential for non-rodent trophectoderm early development. Cell Rep. 2022;41(8):111644. doi:10.1016/j.celrep.2022.111644
31.Li W, Yang S, Liu H, et al. Identification of key LncRNAs and mRNAs associated with intramuscular fat in pig via WGCNA. BMC Genomics. 2025;26(1):233. Published 2025 Mar 11. doi:10.1186/s12864-025-11427-x
32.Liu J, Xu L, Ding X, Ma Y. Genome-Wide Association Analysis of Reproductive Traits in Chinese Holstein Cattle. Genes (Basel). 2023;15(1):12. Published 2023 Dec 20. doi:10.3390/genes15010012
33.Topno NA, Kesarwani V, Kushwaha SK, et al. Non-Synonymous Variants in Fat QTL Genes among High- and Low-Milk-Yielding Indigenous Breeds. Animals (Basel). 2023;13(5):884. Published 2023 Feb 28. doi:10.3390/ani13050884
34.Xiong H, Zhang Y, Zhao Z. Investigation of single nucleotide polymorphisms in differentially expressed genes and proteins reveals the genetic basis of skeletal muscle growth differences between Tibetan and Large White pigs. Anim Biosci. 2024;37(12):2021-2032. doi:10.5713/ab.24.0135
35.Sánchez-Ramos R, Trujano-Chavez MZ, Gallegos-Sánchez J, Becerril-Pérez CM, Cadena-Villegas S, Cortez-Romero C. Detection of Candidate Genes Associated with Fecundity through Genome-Wide Selection Signatures of Katahdin Ewes. Animals (Basel). 2023;13(2):272. Published 2023 Jan 13. doi:10.3390/ani13020272
36.Sánchez-Ramos R, Trujano-Chavez MZ, Gallegos-Sánchez J, Becerril-Pérez CM, Cadena-Villegas S, Cortez-Romero C. Detection of Candidate Genes Associated with Fecundity through Genome-Wide Selection Signatures of Katahdin Ewes. Animals (Basel). 2023 Jan 13;13(2):272. doi: 10.3390/ani13020272.
37.Wei Y, Zhu C, He X, Chu M. Hypothalamus Transcriptome Reveals Key lncRNAs and mRNAs Associated with Fecundity in Goats. Animals (Basel). 2025 Mar 6;15(5):754. doi: 10.3390/ani15050754.
38.An ZX, Shi LG, Hou GY, Zhou HL, Xun WJ. Genetic diversity and selection signatures in Hainan black goats revealed by whole-genome sequencing data. Animal. 2024

Jun;18(6):101147. doi: 10.1016/j.animal.2024.101147."
39.Butty AM, Chud TCS, Cardoso DF, et al. Genome-wide association study between copy number variants and hoof health traits in Holstein dairy cattle. J Dairy Sci. 2021;104(7):8050-8061. doi:10.3168/jds.2020-19879
