# Supplementary material for: Integrating selective signature analysis and genome-wide association study to identify candidate genes associated with litter size in Leizhou goat
Source: Anim Biosci. 2026 Jul 27;39(11):260280. doi: 10.5713/ab.260280 (PMC13740650; doi:10.5713/ab.260280)
Supplement: Supplementary file 6 [file ab-260280-Supplementary-14.pdf]

Supplement 14. Results of KEGG enrichment analysis for the 207 candidate genes identified by GWAS.

Term	ID	Input number	Background number	P-Value	Input
MAPK signaling pathway	chx04010	8	299	0.004205	*RASGRP2|MAP4K2|PDGFD|CACNG8|ELK4|MAPK8|CACNG6|CACNG7*
Dopaminergic synapse	chx04728	5	131	0.005569	*DDC|KCNJ3|MAPK8|ATF6B|PPP2R2B*
Arrhythmogenic right ventricular cardiomyopathy (ARVC)	chx05412	4	82	0.00572	*CACNG8|DES|CACNG6|CACNG7*
Metabolic pathways	chx01100	22	1505	0.008135	*PLCZ1|GPAT4|PI4K2A|ACADL|PPT2|PLA2G7|FAH|RPE|ETNK2|MECR|KYAT3|PYGM|B4GALT7|PHYKPL|MTHFS|GMPPA|CHST9|PIK3C2G|DDC|HOGA1|AGPAT1|CHPF*
Hypertrophic cardiomyopathy (HCM)	chx05410	4	95	0.009335	*CACNG8|DES|CACNG6|CACNG7*
Dilated cardiomyopathy (DCM)	chx05414	4	98	0.010339	*CACNG8|DES|CACNG6|CACNG7*
Adrenergic signaling in cardiomyocytes	chx04261	5	155	0.01084	*CACNG8|ATF6B|CACNG7|CACNG6|PPP2R2B*
Glycosaminoglycan biosynthesis - chondroitin sulfate / dermatan sulfate	chx00532	2	19	0.012933	*CHPF|B4GALT7*
Fatty acid metabolism	chx01212	3	60	0.015458	*MECR|PPT2|ACADL*
Inositol phosphate metabolism	chx00562	3	75	0.027076	*PLCZ1|PIK3C2G|PI4K2A*
Fatty acid elongation	chx00062	2	31	0.03048	*MECR|PPT2*

Apoptosis	chx04210	4	151	0.040298	*TUBA4A*|*MAPK8*|*HRK*|*BCL2A1*
Cardiac muscle contraction	chx04260	3	90	0.04225	*CACNG8*|*CACNG6*|*CACNG7*
Oxytocin signaling pathway	chx04921	4	155	0.043563	*KCNJ3*|*CACNG8*|*CACNG6*|*CACNG7*
