# Supplementary material for: Integrating selective signature analysis and genome-wide association study to identify candidate genes associated with litter size in Leizhou goat
Source: Anim Biosci. 2026 Jul 27;39(11):260280. doi: 10.5713/ab.260280 (PMC13740650; doi:10.5713/ab.260280)
Supplement: Supplementary file 7 [file ab-260280-Supplementary-15.pdf]

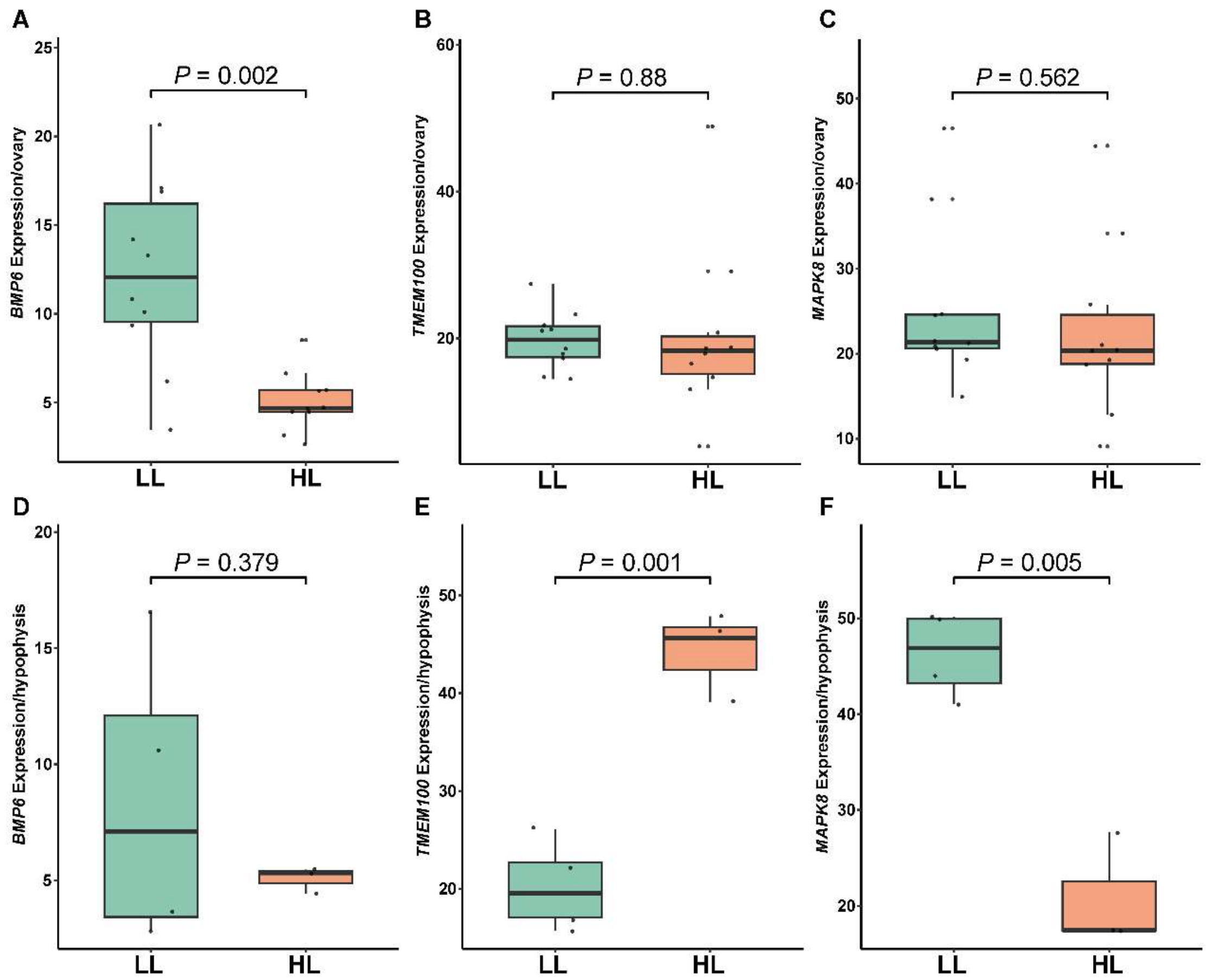

Supplement 15. Differential expression of *BMP6*, *TMEM100*, and *MA PK8* genes in ovarian and pituitary tissues. A. Differential expression of *BMP6* in ovarian tissues between high- and low-fertility groups. B. Differe ntial expression of *TMEM100* in ovarian tissues between high- and low-fe rtility groups. C. Differential expression of *MAPK8* in ovarian tissues betw een high- and low-fertility groups. D. Differential expression of *BMP6* in h ypophysis tissues between high- and low-fertility groups. E. Differential ex pression of *TMEM100* in hypophysis tissues between high- and low-fertilit y groups. F. Differential expression of *MAPK8* in hypophysis tissues betw een high- and low-fertility groups.
