# Supplementary material for: Integrating selective signature analysis and genome-wide association study to identify candidate genes associated with litter size in Leizhou goat
Source: Anim Biosci. 2026 Jul 27;39(11):260280. doi: 10.5713/ab.260280 (PMC13740650; doi:10.5713/ab.260280)
Supplement: Supplementary file 8 [file ab-260280-Supplementary-2.pdf]

**Supplement 2.** Distribution of SNPs identified in Leizhou goats within various genomic regions annotated by ANNOVAR.

| **Category** | **SNP Count** |
|---|---|
| intergenic | 13,616,229 |
| intronic | 5,764,891 |
| ncRNA_intronic | 861,248 |
| upstream | 150,156 |
| downstream | 146,512 |
| exonic | 141,724 |
| UTR3 | 68,141 |
| ncRNA_exonic | 33,680 |
| UTR5 | 16,083 |
| upstream;downstream | 2,973 |
| splicing | 564 |
| ncRNA_splicing | 153 |
| exonic;splicing | 81 |
| UTR5;UTR3 | 64 |
| ncRNA_exonic;splicing | 15 |
