# Supplementary material for: Integrating selective signature analysis and genome-wide association study to identify candidate genes associated with litter size in Leizhou goat
Source: Anim Biosci. 2026 Jul 27;39(11):260280. doi: 10.5713/ab.260280 (PMC13740650; doi:10.5713/ab.260280)
Supplement: Supplementary file 9 [file ab-260280-Supplementary-3.pdf]

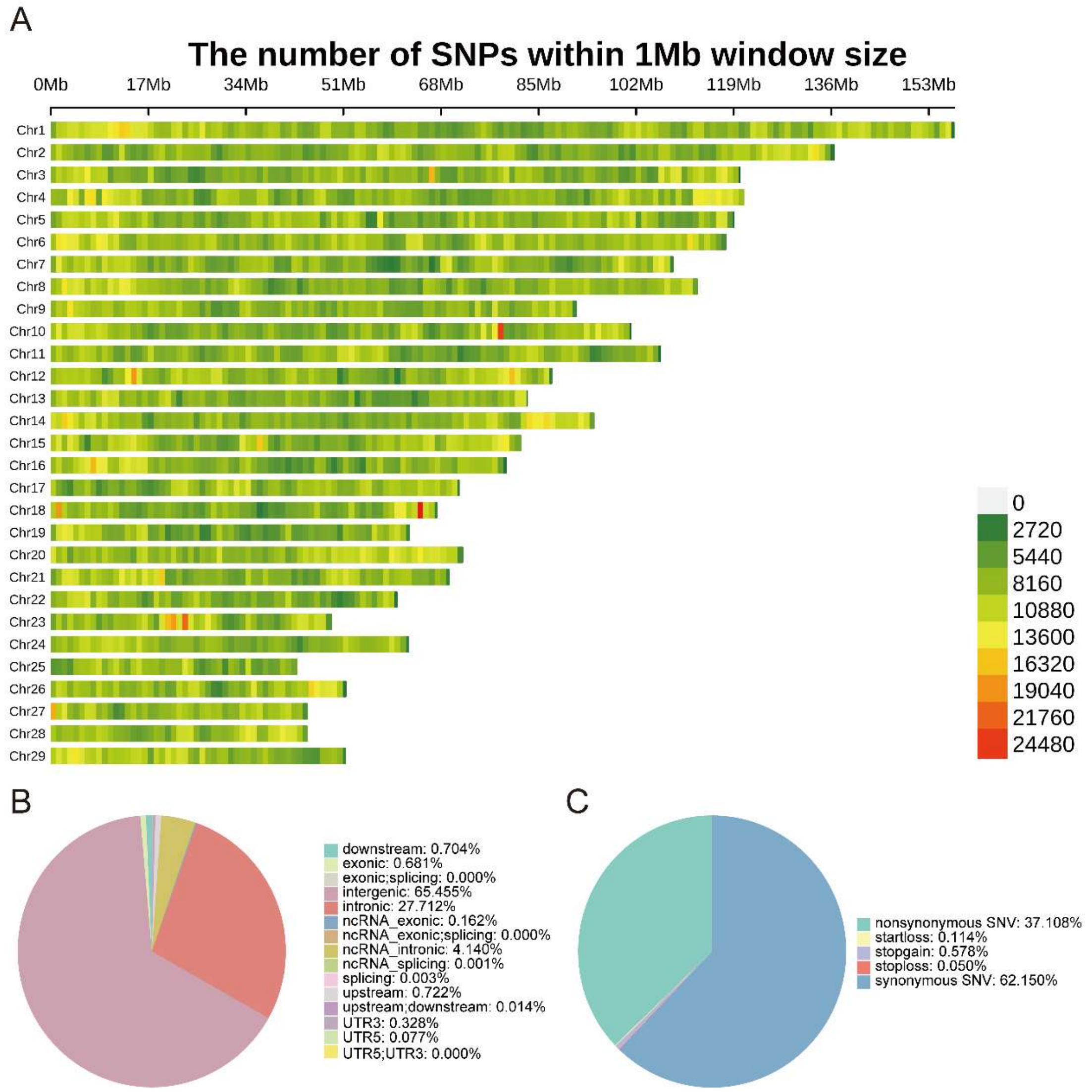

Supplement 3. The distribution of SNPs in Leizhou goat chromosomes and their genomic region annotation. A. The distribution of SNPs on 29 chromosomes, with the y-axis representing 1-29 chromosomes and the x-axis representing every 1Mb window on the chromosome. Different colors indicate the number of SNPs contained in each 1Mb window. B. Annotation status of SNPs in genomic regions. C. SNP variants located in exon regions.
