# Supplementary material for: Integrating selective signature analysis and genome-wide association study to identify candidate genes associated with litter size in Leizhou goat
Source: Anim Biosci. 2026 Jul 27;39(11):260280. doi: 10.5713/ab.260280 (PMC13740650; doi:10.5713/ab.260280)
Supplement: Supplementary file 10 [file ab-260280-Supplementary-4.pdf]

Supplement 4. Distribution of SNPs identified in Leizhou goats within various exonic regions annotated by ANNOVAR.

**Category**	**SNP Count**
synonymous SNP	88,132
nonsynonymous SNP	52,621
stopgain	819
startloss	162
stoploss	71
