# Supplementary material for: Integrating selective signature analysis and genome-wide association study to identify candidate genes associated with litter size in Leizhou goat
Source: Anim Biosci. 2026 Jul 27;39(11):260280. doi: 10.5713/ab.260280 (PMC13740650; doi:10.5713/ab.260280)
Supplement: Supplementary file 11 [file ab-260280-Supplementary-5.pdf]

Supplement 5. Autosomal regions identified as candidate selection sweeps by comparing genetic variants between 30 high litter size and 30 low litter size in Leizhou goats. The statistics significant regions for F_{ST}/log2-ratio (θπ, HL/θπ, LL)/XP-EHH in two main groups were calculated separately. The functions related to these genes were annotated through literature mining.

No.	Chrom	Start(bp)	End(bp)	Max(F_{ST})	Max log2-ratio（θπ, HL/θπ, LL）	Max XP-EHH	Protein-coding gene	Related Phenotype
1	1	16,410,001	16,455,000	0.072	0.907	1.79	-	-
2	1	26,450,001	26,490,000	0.051	0.493	1.71	-	-
3	1	29,380,001	29,420,000	0.048	0.679	1.69	-	-
4	1	31,435,001	31,915,000	0.042	0.838	2.04	*CADM2*	1.Growth of goat[1]<br>2.Body size of sheep[2]<br>3.Fat development of pig[3]
5	1	42,655,001	42,685,000	0.053	0.899	2.07	*CPOX,LOC102168768*	Number of ribs in sheep[4]
6	1	47,840,001	47,905,000	0.052	0.899	2.5	-	-
7	1	49,235,001	49,255,000	0.035	0.824	1.51	*ALCAM, LOC102170735*	Immune of cattle[5]
8	1	52,565,001	52,590,000	0.035	0.302	1.72	*CD47*	-
9	1	54,235,001	54,260,000	0.039	1.041	1.51	-	-
10	1	69,995,001	70,015,000	0.042	0.682	1.46	*LRCH3*	-

11	1	76,980,001	77,035,000	0.032	0.877	2.15	*P3H2*	-
12	1	81,035,001	81,060,000	0.049	0.318	2.41	*IGF2BP2*	Endometrial function of sheep[6]
13	1	83,615,001	83,640,000	0.039	0.505	2.12	*MCF2L2,LAMP3,LOC106501989*	-
14	1	84,000,001	84,020,000	0.030	0.384	1.78	-	-
15	1	86,035,001	86,515,000	0.076	0.901	2.77	*CCDC39,TTC14*	Litter size of goat[7]
16	1	91,165,001	91,200,000	0.045	0.418	2.03	*NAALADL2*	Tolerance to heat[8]
17	1	94,570,001	94,595,000	0.031	0.370	1.71	*TNFSF10,GHSR*	Growth of goat[9]
18	1	97,350,001	97,385,000	0.055	0.502	1.74	-	-
19	1	98,295,001	98,325,000	0.034	0.643	2.17	-	-
20	1	98,385,001	98,525,000	0.057	1.179	2.64	*GOLIM4*	Feed efficiency of cattle[10]
21	1	110,725,001	110,760,000	0.040	0.743	3.26	*KCNAB1*	Somatic cell score of cattle[11]
22	1	112,185,001	112,275,000	0.041	0.619	2.84	-	-
23	1	113,410,001	113,505,000	0.100	1.233	2.28	-	-
24	1	117,980,001	118,005,000	0.034	0.434	1.55	*COMMD2*	-
25	1	119,375,001	119,745,000	0.082	0.723	2.28	*SNORA70*	-
26	1	133,865,001	133,885,000	0.032	0.593	1.76	*EPHB1*	-
27	1	134,850,001	134,875,000	0.042	0.468	1.63	*RYK,SLCO2A1*	-
28	1	135,050,001	135,075,000	0.037	0.627	1.67	*RAB6B*	Birth weight of sheep[12]

29	1	135,845,001	135,910,000	0.076	0.893	2.98	*TMEM108*	Body size of pig[13]
30	1	137,455,001	137,475,000	0.033	0.477	1.68	-	-
31	1	139,345,001	139,375,000	0.101	0.457	1.61	*B3GALT5*	Reproduction of cattle[14]
32	1	140,220,001	140,240,000	0.040	0.301	1.69	-	-
33	1	140,345,001	140,380,000	0.059	1.008	1.83	*RPS3A*	-
34	1	141,560,001	141,630,000	0.041	0.617	2.7	*RIPK4*	-
35	1	148,435,001	148,480,000	0.088	0.672	3.84	*DOP1B*	-
36	1	148,880,001	148,910,000	0.040	0.533	2.17	*HLCS,SIM2*	Meat quality of cattle[15]
37	1	150,685,001	150,705,000	0.032	0.509	1.95	*ERG,SNORA70*	-
38	1	151,220,001	151,250,000	0.045	0.692	1.56	-	-
39	2	8,435,001	8,480,000	0.072	0.824	1.49	*LDLRAP1*	-
40	2	10,580,001	10,675,000	0.069	0.772	1.7	*EYA3*	Meat quality of cattle[16]
41	2	62,015,001	62,085,000	0.084	0.543	2.66	-	-
42	2	62,710,001	62,730,000	0.049	0.301	1.82	*NIFK,TSN*	-
43	2	64,975,001	65,000,000	0.037	0.338	1.7	-	-
44	2	68,345,001	68,365,000	0.036	0.337	1.68	*DPP10*	-
45	2	71,900,001	71,920,000	0.032	0.431	1.53	*NCKAP5*	Reproduction of cattle[17]
46	2	102,060,001	102,165,000	0.071	0.708	1.77	*IFIH1,GCA,KCNH7*	1.Growth of sheep[18]<br>2.Litter size of goat[19]
47	2	105,995,001	106,020,000	0.034	0.558	1.92	*SCN1A*	Spermatozoa of cattle[20]
48	3	9,360,001	9,390,000	0.033	0.475	2.22	*GJB5*	-

49	3	39,580,001	39,710,000	0.054	0.588	2.46	*UBE2U, CACHD1*	Meat quality of cattle[21]
50	3	46,550,001	46,570,000	0.054	0.307	1.44	*PTGER3*	Reproduction of cattle[22]
51	3	53,140,001	53,315,000	0.050	0.506	1.82	-	-
52	3	58,485,001	59,110,000	0.128	0.533	1.93	*ADGRL2*	1.Feed efficiency of pig[23]<br>2.Body weight[24]
53	3	59,550,001	59,940,000	0.047	0.790	2.34	-	-
54	3	61,865,001	61,895,000	0.047	0.392	2.64	*SSX2IP, LPAR3*	Meat quality of cattle[25]
55	3	65,170,001	65,190,000	0.044	0.329	2.22	-	-
56	3	66,790,001	66,830,000	0.062	0.591	1.85	-	-
57	3	67,840,001	67,880,000	0.064	0.389	1.68	*ZNF326, BARHL2*	-
58	3	76,730,001	76,755,000	0.053	0.312	1.55	*PLPPR4, PALMD*	Phospholipid metabolism[26]
59	3	82,325,001	82,365,000	0.091	0.674	2.06	-	-
60	3	83,130,001	83,245,000	0.094	0.458	1.83	-	-
61	3	92,480,001	92,520,000	0.046	0.836	1.71	*TSPAN2, NGF*	-
62	3	95,360,001	95,455,000	0.093	1.102	1.93	*SPAG17*	1.Litter size of sheep[27]<br>2.Body size of goat[28]
63	3	103,445,001	103,470,000	0.056	0.680	1.68	*ILF2, SNAPIN,NPR1*	-
64	3	108,860,001	108,880,000	0.030	0.575	1.76	*SPTA1*	Fatty acid composition of goat[29]
65	3	110,915,001	110,965,000	0.074	0.623	1.54	*SLAMF1*	Immunity of cattle[30]

66	3	111,710,001	111,740,000	0.045	1.016	1.71	*NECTIN4,KLHDC9*	-
67	4	5,415,001	5,445,000	0.041	0.446	2.16	*GALNTL5, PRKAG2*	-
68	4	9,175,001	9,795,000	0.056	0.770	3.12	-	-
69	4	10,845,001	10,890,000	0.054	0.580	1.9	-	-
70	4	13,190,001	13,230,000	0.040	0.436	2.42	*OR2A2*	Reproduction of cattle[31]
71	4	16,100,001	16,120,000	0.045	0.303	2.44	-	-
72	4	21,510,001	21,545,000	0.065	0.758	2.01	*CALD1*	-
73	4	43,175,001	43,200,000	0.030	0.344	1.6	*CAMK2B, NUDCD3*	Meat quality of goat[32]
74	4	47,705,001	47,725,000	0.037	0.418	1.6	-	-
75	4	50,730,001	50,755,000	0.062	0.413	2.63	*SNX10*	-
76	4	51,235,001	51,280,000	0.059	0.606	2	*SKAP2,HOXA1,HOXA2,HOXA3*	1.Growth of pig[33]<br>2.Reproduction of sheep[34]
77	4	51,330,001	51,395,000	0.068	0.441	2.2	*HOXA9, HOXA10,HOXA11,HOXA13*	1.Reproduction of sheep[35]<br>2.Intramusular fat of pig[36]<br>3.Litter size of pig[37]<br>4.Fat tail formation of sheep[38]<br>5.Gestation length of pig[39]

								6.Reproduction of sheep[40]
78	4	62,315,001	62,345,000	0.038	0.484	2.35	-	-
79	4	64,350,001	64,375,000	0.032	0.486	1.46	*LSMEM1*	-
80	4	68,040,001	68,090,000	0.060	1.182	1.83	*TFEC, TES*	-
81	4	83,060,001	83,100,000	0.042	0.563	1.93	*PCLO, SEMA3E*	Calving performance[41]
82	5	4,095,001	4,125,000	0.043	0.660	1.84	*ATXN7L3B*	-
83	5	7,000,001	7,030,000	0.038	0.780	1.57	*NAV3*	-
84	5	9,295,001	9,320,000	0.033	0.730	2.24	-	-
85	5	18,835,001	18,865,000	0.039	0.483	1.67	*KITLG, DUSP6*	Litter size of goat[42]
86	5	39,525,001	39,645,000	0.051	0.452	2.28	*CNTN1, LRRK2*	Growth of pig[43]
87	5	42,600,001	42,700,000	0.092	0.607	4.32	*KCNMB4,CNOT2*	Muscle development of cattle[44]
88	5	49,140,001	49,755,000	0.051	0.551	2.86	*SRGAP1, RXYLT1, AVPR1A, PPM1H*	Meat quality of goat[45]
89	5	50,780,001	50,800,000	0.040	0.715	1.59	-	-
90	5	69,105,001	69,130,000	0.055	0.712	1.5	*CRY1, BTBD11*	-
91	5	78,850,001	78,875,000	0.038	0.452	1.45	*FAR2*	Feed conversion ratio of pig[46]
92	5	87,440,001	87,470,000	0.037	0.445	2.07	*SLCO1A2*	Reproduction of cattle[47]
93	5	104,515,001	104,535,000	0.058	0.440	1.65	-	-
94	5	104,705,001	104,725,000	0.039	0.567	1.57	*CCND2*	Growth of cattle[48]

95	5	107,970,001	107,990,000	0.033	0.319	1.42	*CECR2,*	-
96	5	110,465,001	110,505,000	0.045	1.754	1.58	*TNRC6B*	Muscle development[49]
97	5	111,175,001	111,195,000	0.049	0.485	1.41	*EP300*	Meat quality of goat[50]
98	5	111,700,001	111,740,000	0.041	0.775	2	*MEI1*	Reproduction of goat[51]
99	5	114,835,001	114,870,000	0.039	0.398	2.01	*FBLN1*	Slaughter of cattle[52]
100	6	10,820,001	11,060,000	0.073	0.522	3.32	*NDST4*	-
101	6	13,910,001	13,935,000	0.041	0.508	1.57	-	-
102	6	38,280,001	38,310,000	0.093	1.422	1.49	*LCORL*	Growth of cattle[53]
103	6	60,685,001	60,790,000	0.039	1.505	2.72	*UCHL1, LIMCH1*	Mammalian spermatogenesis of goat[54]
104	6	67,495,001	67,525,000	0.044	0.740	2.26	*TEC, SLAIN2*	-
105	6	94,895,001	95,075,000	0.044	1.102	2.25	*ANTXR2*	Reproduction of goat[55]
106	6	102,850,001	102,875,000	0.031	0.468	1.8	*SPARCL1, DSPP*	Intramuscular fat[56]
107	6	103,040,001	103,060,000	0.041	0.556	1.57	*DMP1, PPP2R2C*	Growth of cattle[57]
108	6	108,810,001	108,835,000	0.049	0.351	1.47	*RAB28*	-
109	6	111,000,001	111,025,000	0.031	0.405	1.86	*CC2D2A*	Litter size of pig[58]
110	6	116,225,001	116,245,000	0.030	0.661	1.76	*ZFYVE28*	-
111	7	16,525,001	16,610,000	0.080	0.530	1.69	*MCTP1, SLF1*	1.Body size of sheep[59]<br>2.Udder conformation of cattle[60]
112	7	18,265,001	18,350,000	0.085	0.357	2.55	*NR2F1*	-

113	7	18,340,001	18,360,000	0.037	0.589	1.51	-	-
114	7	31,830,001	31,930,000	0.040	0.575	1.5	-	-
115	7	34,160,001	34,185,000	0.032	0.364	1.83	-	-
116	7	66,345,001	66,415,000	0.068	0.496	1.83	*ATP8B3,TCF3*	-
117	7	66,485,001	66,510,000	0.034	0.312	1.52	*UQCR11*	Cold adaptation of cattle[61]
118	7	70,615,001	70,690,000	0.065	0.732	2.04	*ZFP62, MGAT1, OR2Y1*	Body weight of cattle[62]
119	7	77,015,001	77,040,000	0.043	0.412	1.57	*PRR16*	-
120	7	84,575,001	84,595,000	0.030	0.539	2.33	-	-
121	7	89,010,001	89,030,000	0.030	0.435	2.03	*SEPTIN8, MKNK2*	-
122	7	91,160,001	91,195,000	0.056	0.343	1.66	*FEM1A*	-
123	7	94,940,001	94,965,000	0.054	0.524	1.49	*ATG4D,KRI1,AP1M2,CDKN2D*	-
124	7	95,910,001	95,935,000	0.037	0.512	2.62	*INSR*	Milk production of goat[63]
125	7	99,155,001	99,175,000	0.034	0.342	1.58	-	-
126	7	99,705,001	99,735,000	0.059	0.407	1.79	-	-
127	7	99,995,001	100,030,000	0.054	0.400	2.16	-	-
128	7	102,745,001	102,775,000	0.042	0.580	1.49	-	-
129	7	103,785,001	103,805,000	0.031	0.421	1.41	*RAB3A*	-
130	7	106,480,001	106,500,000	0.030	0.383	1.65	*ZNF354C*	-
131	7	106,530,001	106,550,000	0.033	0.445	1.59	*ADAMTS2*	Body size of goat[64]

132	8	10,555,001	10,590,000	0.058	0.433	2.52	*NUGGC, ELP3*	Litter size of goat[65]
133	8	26,435,001	26,495,000	0.076	0.748	2.33	*SH3GL2*	-
134	8	42,610,001	42,655,000	0.069	0.370	1.47	-	-
135	8	44,035,001	44,095,000	0.043	0.553	2.16	*DOCK8*	Milk yield of cattle[66]
136	8	66,560,001	66,585,000	0.031	0.547	1.86	*LPL*	-
137	8	105,790,001	105,810,000	0.041	1.023	2.1	*ASTN2*	Long-haired of yaks[67]
138	8	111,885,001	111,930,000	0.062	0.895	2.2	*SNTG2, TPO*	-
139	8	112,260,001	112,310,000	0.048	0.385	2.61	*TMEM18, ALKAL2*	Growth of cattle[68]
140	8	112,510,001	112,550,000	0.051	0.662	1.69	*SH3YL1, FAM110C*	-
141	9	5,565,001	5,585,000	0.052	0.553	1.48	*MEI4*	-
142	9	39,480,001	39,810,000	0.035	0.403	2.05	*MMS22L*	Teat-Shape Conformation of cattle[69]
143	9	40,270,001	40,305,000	0.038	0.849	2.38	*GPR63*	-
144	9	57,750,001	57,775,000	0.044	0.422	2.01	*TAAR8*	-
145	9	59,080,001	59,115,000	0.032	0.362	1.44	*EYA4, TCF21*	Muscle development of cattle[70]
146	9	75,980,001	76,005,000	0.042	0.371	2.17	*CCDC170, ESR1*	Reproduction of goat[71]
147	9	76,695,001	76,715,000	0.034	0.641	2.21	*SYNE1*	1.Reproduction of sheep[72]<br>2.Meat quality of sheep[73]
148	9	78,950,001	78,985,000	0.078	0.473	2.3	*TIAM2*	-

149	9	81,625,001	81,645,000	0.031	0.421	2.77	-	-
150	9	83,355,001	83,380,000	0.038	0.409	1.66	*PNLDC1,TCP1,MRPL18*	-
151	10	20,980,001	21,000,000	0.040	0.445	1.45	*ADAM21,COX16*	-
152	10	47,610,001	47,630,000	0.055	0.301	1.6	*UNC13C*	-
153	10	84,895,001	84,915,000	0.031	0.327	1.47	*RPLP1*	-
154	10	85,110,001	85,155,000	0.073	1.003	2.99	*GLCE*	Milk yield of cattle[74]
155	10	97,755,001	97,780,000	0.061	0.509	1.81	*TRIM36,KCNN2*	-
156	11	965,001	1,055,000	0.101	0.776	2.67	*ANAPC1,BCL2L11*	-
157	11	5,010,001	5,030,000	0.032	0.345	2.44	*AFF3*	-
158	11	13,505,001	13,550,000	0.055	0.831	2.03	*FIGLA,ADD2*	Folliculogenesis[75]
159	11	13,640,001	13,680,000	0.046	0.584	1.66	-	-
160	11	18,650,001	18,680,000	0.039	0.797	3.35	*CRIM1*	Mammary gland morphology of cattle[76]
161	11	19,470,001	19,560,000	0.082	1.653	2.04	*CEBPZOS,SULT6B1,CEBPZ,NDUFAF7,PRKD3*	Feed efficiency of pig[77]
162	11	19,705,001	19,725,000	0.035	0.513	1.52	*QPCT,CDC42EP3*	-
163	11	22,665,001	22,685,000	0.071	0.591	1.44	*SLC8A1*	Tail length of cattle[78]
164	11	23,830,001	23,850,000	0.033	0.420	1.9	-	-
165	11	23,960,001	23,980,000	0.035	0.469	1.89	-	-
166	11	31,760,001	31,785,000	0.054	0.380	1.6	-	-
167	11	52,545,001	52,565,000	0.042	0.355	1.54	-	-

168	11	65,685,001	65,765,000	0.060	0.980	2.27	-	-
169	11	74,100,001	74,130,000	0.042	1.172	1.7	*CENPO,ADCY3*	Follicular development of mice[79]
170	11	82,830,001	82,850,000	0.033	0.552	1.48	-	-
171	11	91,345,001	91,505,000	0.056	0.558	2.22	-	-
172	11	99,390,001	99,410,000	0.035	0.370	1.44	*IER5L,NTMT1*	-
173	11	102,860,001	102,880,000	0.034	0.428	1.94	*CAMSAP1*	Milk urea nitrogen of cattle[80]
174	12	14,170,001	14,190,000	0.050	0.798	1.78	-	-
175	12	14,445,001	14,480,000	0.126	1.954	1.64	-	-
176	12	15,415,001	15,470,000	0.175	1.348	2.3	-	-
177	12	15,755,001	15,785,000	0.123	1.044	2.16	-	-
178	12	16,185,001	16,205,000	0.031	0.303	1.75	-	-
179	12	23,390,001	23,420,000	0.059	0.406	1.8	*SLITRK5*	-
180	12	24,465,001	24,500,000	0.038	0.530	2.39	*SLITRK5*	Milk yield of cattle[81]
181	12	25,395,001	25,415,000	0.031	0.679	2.29	*SLITRK6*	-
182	12	27,570,001	27,600,000	0.056	0.562	1.95	*SLITRK1*	-
183	12	33,160,001	33,195,000	0.081	0.615	2.2	*SLAIN1*	-
184	12	34,860,001	34,885,000	0.034	0.344	1.48	*KCTD12*	-
185	12	34,965,001	34,985,000	0.033	0.617	1.45	-	-
186	12	35,260,001	35,370,000	0.053	1.746	2.85	*LMO7*	-

187	12	35,720,001	35,740,000	0.030	0.307	1.42	*LMO7,UCHL3*	Muscle development[82]
188	12	41,175,001	41,460,000	0.044	0.435	1.93	-	-
189	12	45,975,001	45,995,000	0.050	1.232	1.65	-	-
190	12	76,545,001	76,610,000	0.049	0.616	2.09	-	-
191	12	78,265,001	78,285,000	0.066	0.616	1.45	-	-
192	13	9,200,001	9,225,000	0.032	0.590	1.56	-	-
193	13	34,445,001	34,470,000	0.037	0.378	1.56	*JCAD*	-
194	13	50,740,001	50,850,000	0.058	0.711	4.02	*CDC25B,CENPB,SPEF1,ADISSP,HSPA12B,SIGLEC1*	1.Reproduction of goat[83]<br>2.Heat stress of goat[84]
195	13	50,870,001	50,890,000	0.040	0.328	1.8	*ADAM33,GFRA4*	-
196	13	51,380,001	51,440,000	0.111	0.585	2.16	*FASTKD5,LZTS3,UBOX5*	Immunity of sheep[85]
197	13	58,185,001	58,300,000	0.056	0.953	2.04	*BMP7*	Reproduction of pig;sheep[86]
198	13	64,095,001	64,135,000	0.095	0.907	2.24	*EIF6,MMP24,FAM83C*	-
199	13	67,385,001	67,410,000	0.038	0.457	1.62	*DHX35*	-
200	13	71,615,001	71,635,000	0.032	1.489	1.47	-	-
201	13	75,650,001	75,675,000	0.035	0.447	1.7	*NCOA3*	Reproduction of cattle[87]
202	13	81,970,001	81,990,000	0.031	0.321	2.08	-	-
203	14	7,185,001	7,240,000	0.037	0.383	3.16	*MMP16*	-
204	14	10,510,001	10,685,000	0.047	0.429	2.47	*RUNX1T1,TRIQK*	Muscle development[88]

205	14	13,035,001	13,125,000	0.091	1.648	2.34	*PLEKHF2,CFAP418*	-
206	14	22,940,001	22,965,000	0.039	1.054	1.97	-	-
207	14	27,285,001	27,305,000	0.031	0.601	1.74	*PKHD1L1*	-
208	14	36,915,001	36,945,000	0.080	0.640	2.81	*COLEC10*	-
209	14	42,670,001	42,745,000	0.061	0.677	2.6	-	-
210	14	52,110,001	52,160,000	0.082	0.635	2.3	*ARMC1*	Somatic cell score of cattle[89]
211	14	57,425,001	57,445,000	0.043	0.331	2.14	*CYP7A1,UBXN2B*	Milk yield of cattle[90]
212	14	68,410,001	68,430,000	0.033	0.476	1.89	-	-
213	14	73,665,001	73,705,000	0.053	0.372	1.68	*SLA*	-
214	14	80,145,001	80,355,000	0.105	0.913	3.94	*ADGRB1,TSNARE1*	1.Milk fatty acid composition of cattle[91] 2.Heat stress of cattle[92]
215	14	89,910,001	89,940,000	0.055	0.362	1.96	*ADGRB3*	1.Litter size of sheep[93]
216	14	91,460,001	91,500,000	0.081	0.520	2.72	*SMAP1*	-
217	15	1,340,001	1,365,000	0.051	0.433	1.56	-	-
218	15	12,140,001	12,160,000	0.034	0.325	1.53	-	-
219	15	13,625,001	13,655,000	0.055	0.744	1.98	-	-
220	15	18,530,001	18,550,000	0.035	0.357	1.7	*FBXO3*	-
221	15	33,465,001	33,510,000	0.141	0.466	2.05	-	-
222	15	46,875,001	46,895,000	0.038	0.316	2.31	*PLEKHA7*	-

223	15	80,230,001	80,285,000	0.070	1.119	2.39	-	-
224	15	80,695,001	80,715,000	0.048	1.694	1.7	*GRIA4,MSANTD4*	1.Early weaning of goat[94]<br>2.Heat stress of cattle[95]
225	16	4,810,001	4,835,000	0.047	0.878	2.4	*CR2*	-
226	16	11,650,001	11,685,000	0.044	0.639	2.39	*RGS21,RGS18*	Reproduction of cattle[96]
227	16	12,820,001	12,855,000	0.073	0.415	2.13	-	-
228	16	40,890,001	41,025,000	0.059	0.900	2.47	*ANGPTL7,MTOR*	Reproduction of goat[97]
229	16	41,425,001	41,460,000	0.099	0.508	1.53	*CASZ1*	-
230	16	46,115,001	46,140,000	0.046	0.561	1.46	*NPHP4*	-
231	16	57,545,001	57,565,000	0.054	0.569	1.45	*SEC16B*	-
232	16	61,340,001	61,380,000	0.046	0.509	2.59	*CACNA1E*	Litter size in sheep[98]
233	16	62,860,001	62,880,000	0.051	0.326	1.94	*LAMC2*	-
234	16	63,235,001	63,255,000	0.039	1.617	1.79	*NCF2*	Immunity[99]
235	16	71,505,001	71,530,000	0.077	0.444	1.62	*HHAT*	-
236	17	10,120,001	10,145,000	0.046	0.304	1.89	-	-
237	17	10,650,001	10,675,000	0.032	0.387	1.79	*RBM19,TBX5*	Mammary gland morphology of cattle[100]
238	17	34,950,001	34,975,000	0.038	0.462	1.55	*FSTL5,TRPC3*	-
239	17	47,790,001	47,820,000	0.037	0.446	1.75	-	-
240	17	50,555,001	50,575,000	0.032	0.372	1.47	*PCDH18*	Feed efficiency of cattle[101]

241	17	60,130,001	60,160,000	0.046	0.638	2.15	*EDNRA*	Meat development of goat[102]
242	17	61,920,001	62,115,000	0.089	1.037	3.08	*IQCM*	-
243	17	64,480,001	64,770,000	0.057	0.731	2.36	*FHIP1A,GATB*	Heat stress of cattle[103]
244	17	69,660,001	69,695,000	0.076	0.535	1.87	*CPE*	-
245	18	1,155,001	1,175,000	0.057	1.258	1.47	-	-
246	18	17,715,001	17,755,000	0.121	0.515	2.73	*SALL1*	Kidney development[104]
247	18	24,115,001	24,135,000	0.033	0.976	1.73	*IRX3*	Litter size of pig[105]
248	18	25,895,001	25,970,000	0.052	0.987	1.94	*CES5A*	-
249	18	26,960,001	27,015,000	0.043	0.387	2.51	*ADGRG3,ADGRG1*	Immune of goat[106]
250	18	30,330,001	30,355,000	0.082	0.332	1.72	-	-
251	18	30,790,001	30,840,000	0.044	0.846	2.09	*CDH8*	Flight reaction of cattle[107]
252	18	31,720,001	31,760,000	0.069	0.742	2.04	-	-
253	18	34,835,001	34,855,000	0.030	0.353	1.59	-	-
254	18	39,500,001	39,520,000	0.043	0.303	1.95	*ZFHX3*	Milk yield of cattle[108]
255	18	58,180,001	58,200,000	0.032	0.557	1.85	-	-
256	18	59,340,001	59,485,000	0.064	0.959	2.03	-	-
257	18	62,855,001	62,885,000	0.071	0.805	2.12	-	-
258	19	6,065,001	6,135,000	0.042	0.551	2.37	-	-
259	19	6,930,001	6,955,000	0.033	0.485	1.98	*NOG*	-

260	19	7,510,001	7,540,000	0.047	0.377	2.44	*SCPEP1,AKAP1*	-
261	19	51,365,001	51,605,000	0.097	0.664	3.04	*RPTOR,NPTX1,ENDOV,RNF213*	1.Litter size in sheep[109] 2.Reproduction of sheep[110]
262	19	55,530,001	55,565,000	0.041	0.430	1.78	*JPT1,NT5C,ARMC7,SLC16A5*	Growth of goat[111]
263	20	43,135,001	43,725,000	0.070	1.014	3.6	-	-
264	20	50,440,001	50,465,000	0.081	0.364	1.61	-	-
265	20	51,610,001	51,655,000	0.065	0.482	1.65	*CDH12*	-
266	20	54,325,001	54,360,000	0.054	0.754	1.62	-	-
267	20	57,645,001	57,665,000	0.035	0.432	1.67	-	-
268	20	58,635,001	58,660,000	0.036	0.463	1.69	*TRIO*	1.Reproduction of cattle[112]
269	20	60,450,001	60,865,000	0.073	1.150	2.86	-	-
270	20	62,530,001	62,550,000	0.030	0.461	2.74	*DAP*	-
271	20	62,680,001	62,720,000	0.038	0.505	2.78	*ANKRD33B*	-
272	20	63,030,001	65,065,000	0.045	0.926	2.66	*ATPSCKMT,MTRR*	-
273	20	66,405,001	66,425,000	0.032	0.349	2.15	*TENT4A*	-
274	21	4,130,001	4,150,000	0.037	0.661	1.56	*GABRG3*	Litter size of sheep[113]
275	21	6,005,001	6,035,000	0.040	0.461	2.1	*ADAMTS17,LYSMD4*	Body size of cattle[114]

276	21	16,825,001	16,845,000	0.033	0.322	1.67	*AGBL1*	1.Litter size of sheep[115] 2.Intramuscular fat deposition[116]
277	21	18,730,001	18,760,000	0.038	0.429	1.88	*NTRK3,MRPL46*	1.Litter size of goat[117] 2.Muscle development[118]
278	21	26,240,001	26,270,000	0.078	0.590	2.05	-	-
279	21	33,395,001	33,425,000	0.033	0.747	1.82	*CYP1A1*	-
280	21	33,525,001	33,560,000	0.045	0.879	1.6	*ARID3B*	-
281	21	33,970,001	34,000,000	0.046	0.455	1.71	*ISLR,ISLR2*	Muscle development[119]
282	21	34,185,001	34,240,000	0.043	0.539	2.23	-	-
283	21	40,300,001	42,370,000	0.047	0.728	1.92	*AKAP6*	Litter size of goat[120]
284	21	48,495,001	48,525,000	0.045	0.527	1.89	*SEC23A*	-
285	21	55,405,001	55,435,000	0.091	0.517	2.03	*FRMD5,GPR68*	-
286	21	57,705,001	57,730,000	0.037	0.344	1.61	*UNC79*	-
287	21	68,960,001	68,985,000	0.034	0.801	1.88	-	-
288	21	69,300,001	69,430,000	0.055	0.940	2.76	-	-
289	22	23,450,001	23,475,000	0.041	0.313	1.57	*CNTN4*	Reproduction of cattle[121]
290	22	30,195,001	30,240,000	0.038	0.691	2.14	*FOXP1*	Calving interval of cattle[122]
291	22	51,935,001	51,970,000	0.040	0.655	2.96	*MAP4,DHX30*	-

292	23	2,140,001	2,160,000	0.041	0.354	1.71	*RREB1*	Heat tolerance of cattle[123]
293	23	2,720,001	3,020,000	0.095	0.827	4.47	*TXNDC5,BMP6,BLOC1S5, EEF1E1*	Reproduction of goat[124 125]
294	23	13,400,001	13,425,000	0.070	0.421	1.53	*CDKAL1*	Body weight of goat[126]
295	23	19,185,001	19,210,000	0.039	0.547	1.44	-	-
296	23	23,505,001	23,535,000	0.094	0.528	1.83	-	-
297	23	24,510,001	24,540,000	0.064	0.481	1.97	*IL17F,IL17A*	Reproduction of cattle[127]
298	23	33,815,001	33,870,000	0.041	0.516	2.28	*TSPO2,APOBEC2,UNC5CL*	-
299	24	1,535,001	1,555,000	0.035	0.489	2.17	-	-
300	24	6,215,001	6,240,000	0.032	0.919	1.77	-	-
301	24	7,725,001	7,745,000	0.034	0.323	1.44	*DOK6*	Feed efficiency of pig[128]
302	24	9,560,001	9,600,000	0.046	0.792	1.71	*DSEL*	Fatty acid composition of cattle[129]
303	24	10,870,001	10,990,000	0.038	0.797	2.24	*CDH19,CDH7*	Feed efficiency of pig[130]
304	24	22,690,001	22,710,000	0.055	0.778	1.42	*DTNA,U6*	Reproduction of cattle[131]
305	24	28,465,001	28,500,000	0.039	0.328	2.68	-	-
306	24	38,575,001	38,595,000	0.033	0.453	3.22	*DLGAP1*	Reproduction of cattle[132]
307	24	59,285,001	59,350,000	0.050	0.946	1.57	*MC4R*	Body weight of sheep[133]
308	25	7,735,001	7,770,000	0.041	0.558	1.79	*HAPSTR1*	-
309	25	9,625,001	9,660,000	0.048	0.623	2.01	*CLEC16A*	-

310	25	14,660,001	14,690,000	0.045	0.406	2.7	-	-
311	25	23,450,001	23,475,000	0.047	0.362	2.68	-	-
312	25	31,650,001	31,690,000	0.064	0.533	3.26	-	-
313	25	33,555,001	33,630,000	0.071	0.552	4	*FZD9,FKBP6,TRIM50,NSUN5*	Sow maternal behavior[134]
314	25	36,405,001	36,425,000	0.031	0.353	1.72	-	-
315	25	36,965,001	36,985,000	0.031	0.532	1.55	*MEPCE,PPP1R35*	-
316	26	3,065,001	3,090,000	0.036	0.836	1.45	*MGMT*	-
317	26	4,170,001	4,205,000	0.036	0.358	1.95	-	-
318	26	6,415,001	6,440,000	0.048	0.321	2.38	*ADAM12*	1.Body size of cattle[135] 2.Mammary gland morphology[136]
319	26	30,330,001	30,365,000	0.033	0.435	2.09	-	-
320	26	30,535,001	30,570,000	0.051	0.991	2.04	*CHUK,ERLIN1*	Meat quality of goat[137]
321	26	30,640,001	30,660,000	0.033	0.342	1.55	-	-
322	26	30,690,001	30,795,000	0.080	0.680	3.08	*CPN1,DNMBP*	-
323	26	30,830,001	30,870,000	0.054	0.799	2.82	*ABCC2*	Fat-related of pig[138]
324	26	30,910,001	30,935,000	0.039	0.416	1.81	*CUTC*	-
325	27	10,480,001	10,515,000	0.049	0.908	2.61	*IDO2*	Reproduction of pig[139]
326	27	14,155,001	14,180,000	0.040	0.382	1.43	-	-
327	27	15,140,001	15,160,000	0.030	0.392	2.1	-	-

328	27	29,465,001	29,485,000	0.030	0.377	1.57	*SORBS2*	-
329	27	34,175,001	34,195,000	0.050	0.304	1.95	-	-
330	27	38,330,001	38,360,000	0.044	0.555	1.94	-	-
331	27	38,850,001	38,870,000	0.030	0.303	2.12	*MCPH1*	-
332	27	40,375,001	40,400,000	0.049	0.453	1.55	-	-
333	28	2,980,001	3,000,000	0.031	0.517	1.43	*MAPK8*	1.Reproduction of goat[140]<br>2.Heat stress of goat[141]
334	28	5,545,001	5,570,000	0.039	0.377	2.8	-	-
335	28	18,190,001	18,250,000	0.035	0.726	2.15	*UNC5B*	-
336	28	21,270,001	21,745,000	0.042	0.697	1.72	-	-
337	28	25,120,001	25,145,000	0.054	0.614	1.56	-	-
338	28	43,260,001	43,295,000	0.055	0.436	2.01	*GALNT2,URB2*	-
339	29	6,920,001	6,985,000	0.086	1.037	2.03	*RAB38*	-
340	29	24,795,001	24,820,000	0.032	0.490	1.69	-	

Reference

1.Xu Z, Wang X, Zhang Z, et al. Copy number variation of CADM2 gene revealed its association with growth traits across Chinese Capra hircus (goat) populations. Gene. 2020;741:144519. doi:10.1016/j.gene.2020.144519

2.Jiang J, Cao Y, Shan H, Wu J, Song X, Jiang Y. The GWAS Analysis of Body Size and Population Verification of Related SNPs in Hu Sheep. Front Genet. 2021;12:642552. doi:10.3389/fgene.2021.642552

3.Kumar H, Srikanth K, Park W, et al. Transcriptome analysis to identify long non coding RNA (lncRNA) and characterize their functional role in back fat tissue of pig. Gene. 2019;703:71-82. doi:10.1016/j.gene.2019.04.014

4.Zhang D, Zhang X, Li F, Liu T, Hu Z, Gao N, Yuan L, Li X, Zhao Y, Zhao L, Zhang Y, Xu D, Song Q, Cheng J, Wang W. Whole-genome resequencing identified candidate genes associated with the number of ribs in Hu sheep. Genomics. 2021 Jul;113(4):2077-2084. doi: 10.1016/j.ygeno.

5.Velayudhan SM, Alam S, Yin T, et al. Selective Sweeps in Cattle Genomes in Response to the Influence of Urbanization and Environmental Contamination. Genes (Basel). 2023;14(11):2083. Published 2023 Nov 15. doi:10.3390/genes14112083

6.Deng K, Li X, Liu Z, et al. IGF2BP2 regulates the proliferation and migration of endometrial stromal cells through the PI3K/AKT/mTOR signaling pathway in Hu sheep. J Anim Sci. 2024;102:skae129. doi:10.1093/jas/skae129

7.Wang Z, Pan Y, He L, et al. Multiple morphological abnormalities of the sperm flagella (MMAF)-associated genes: The relationships between genetic variation and litter size in goats. Gene. 2020;753:144778. doi:10.1016/j.gene.2020.144778

8.Aboul-Naga AM, Alsamman AM, Melak S, Nassar AE, Abdelsabour TH, Gamal L, Adley M, Elshafie MH. Genomic analysis association of tolerance to heat stress in subtropical Egyptian goats Raised in hot dry environment. BMC Genomics. 2025 Jun 11;26(1):568. doi: 10.1186/s12864-025-11748-x.

9.Jin Q, Fang XT, Yang L, et al. Novel SNPs of the caprine growth hormone secretagogue receptor (GHSR) gene and their association with growth traits in goats. Biochem Genet. 2010;48(9-10):847-856. doi:10.1007/s10528-010-9366-8

10.Olivieri BF, Mercadante ME, Cyrillo JN, et al. Genomic Regions Associated with Feed Efficiency Indicator Traits in an Experimental Nellore Cattle Population. PLoS One. 2016;11(10):e0164390. Published 2016 Oct 19. doi:10.1371/journal.pone.0164390

11.Lu X, Jiang H, Abdelaziz Adam IA, et al. Investigating Genetic Characteristics of Chinese Holstein Cow's Milk Somatic Cell Score by Genetic Parameter Estimation and Genome-Wide Association. Agriculture. 2023;13(2):267. doi:https://doi.org/10.3390/agriculture13020267

12.Lu Z, Yue Y, Chao Y, et al. Genome-Wide Association Study of Body Weight Traits in Chinese Fine-Wool Sheep. Animals. 2020;10(1):170. doi:https://doi.org/10.3390/ani10010170

13.Banestani, E. S., Esmailizadeh, A., Momen, M., Mehrgardi, A. A., & Mokhtari, M. (2023). Genome-wide association study identifies significant SNP and related genes associated with body size in Yorkshire pigs using latent variable modelling. The Journal of Agricultural Science, 161(4), 599-605.

14.Muansangi L, Tiwari J, Ilayaraja I, Kumar I, Vyas J, Chitra A, Singh SP, Pal P, Gowane G, Mishra AK, Mukherjee A, Mukherjee S. DCMS analysis revealed differential selection signatures in the transboundary Sahiwal cattle for major economic traits. Sci Rep. 2025 May 5;15(1):15685. doi: 10.1038/s41598-025-93021-5.

15.de Las Heras-Saldana S, Chung KY, Lee SH, Gondro C. Gene expression of Hanwoo satellite cell differentiation in longissimus dorsi and semimembranosus. BMC Genomics. 2019;20(1):156. Published 2019 Feb 26. doi:10.1186/s12864-019-5530-7

16.Xia X, Zhang S, Zhang H, et al. Assessing genomic diversity and signatures of selection in Jiaxian Red cattle using whole-genome sequencing data. BMC Genomics. 2021;22(1):43. Published 2021 Jan 9. doi:10.1186/s12864-020-07340-0

17.Tourchi, J. J., Alijani, S., Rafat, S. A., & Abbasi, M. A. (2024). Bayesian-genome-wide association study and post-GWAS on reproductive traits of Holstein dairy cattle. Journal of Dairy Research, 91(2), 146-155.
18.Wang Q, Xu J, Bao M, Wang H, Sun X, Ji D, Wang J, Li Y. Weighted gene co-expression network analysis reveals genes related to growth performance in Hu sheep. Sci Rep. 2024 Jun 6;14(1):13043. doi: 10.1038/s41598-024-63850-x.
19.Wang JJ, Zhang T, Chen QM, Zhang RQ, Li L, Cheng SF, Shen W, Lei CZ. Genomic Signatures of Selection Associated With Litter Size Trait in Jining Gray Goat. Front Genet. 2020 Mar 26;11:286. doi: 10.3389/fgene.2020.00286.
20.Hai C, Wang L, Wang S, et al. Loss of Myostatin Affects m6A Modification but Not Semen Characteristics in Bull Spermatozoa. Int J Mol Sci. 2025;26(2):591. Published 2025 Jan 12. doi:10.3390/ijms26020591
21.Ryu J, Lee C. Genetic association of marbling score with intragenic nucleotide variants at selection signals of the bovine genome. Animal. 2016;10(4):566-570. doi:10.1017/S1751731115002633
22.Nuttinck F, Gall L, Ruffini S, et al. PTGS2-related PGE2 affects oocyte MAPK phosphorylation and meiosis progression in cattle: late effects on early embryonic development. Biol Reprod. 2011;84(6):1248-1257. doi:10.1095/biolreprod.110.088211
23.Silva MRGD, Veroneze R, Marques DBD, Silva DAD, Machado II, Brito LF, Lopes PS. A meta-analysis of genome-wide association studies to identify candidate genes associated with feed efficiency traits in pigs. J Anim Sci. 2025 Jan 4;103:skaf010. doi: 10.1093/jas/skaf010.
24.Kang, H. C., Kim, H. S., Lee, J. B., Yoo, C. K., Choi, T. J., & Lim, H. T. (2018). Analysis of the relationship between the end weight trait and the gene ADGRL2 in purebred landrace pigs using a Genome-wide association study. Korean Journal of Agricultural Science, 45(2), 238-247.
25.Han L, Fu R, Fu B, et al. Integrating metabolomics and transcriptomics to analyze differences in muscle mass and flavor formation in Gayal and yellow cattle. Front Vet Sci. 2025;12:1581767. Published 2025 May 14. doi:10.3389/fvets.2025.1581767
26.Gao J, Gonzalez-Prendes R, Liu Y, Kantanen J, Ginja C, Ghanem N, Kugonza DR, Makgahlela M, Bovenhuis H, Groenen MAM, Crooijmans RPMA. Evidence of early genomic selection in Holstein Friesian across African and European ecosystems. BMC Genomics. 2025 Jul 1;26(1):615. doi: 10.1186/s12864-025-11828-y.
27.Tao L, He X, Wang X, Di R, Chu M. Litter Size of Sheep (Ovis aries): Inbreeding Depression and Homozygous Regions. Genes (Basel). 2021 Jan 18;12(1):109. doi: 10.3390/genes12010109.
28.Zhang S, Jiang E, Wang K, et al. Two Insertion/Deletion Variants within SPAG17 Gene Are Associated with Goat Body Measurement Traits. Animals. 2019;9(6):379. doi:https://doi.org/10.3390/ani9060379"
29.Li C, Zhu J, Shi H, Luo J, Zhao W, Shi H, Xu H, Wang H, Loor JJ. Comprehensive Transcriptome Profiling of Dairy Goat Mammary Gland Identifies Genes and Networks Crucial for Lactation and Fatty Acid Metabolism. Front Genet. 2020 Sep 25;11:878. doi: 10.3389/fgene.2020.00878.

30.Song X, Yao Z, Zhang Z, Lyu S, Chen N, Qi X, Liu X, Ma W, Wang W, Lei C, Jiang Y, Wang E, Huang Y. Whole-genome sequencing reveals genomic diversity and selection signatures in Xia'nan cattle. BMC Genomics. 2024 Jun 5;25(1):559. doi: 10.1186/s12864-024-10463-3.

31.Zhou Y, Connor EE, Wiggans GR, et al. Genome-wide copy number variant analysis reveals variants associated with 10 diverse production traits in Holstein cattle. BMC Genomics. 2018;19(1):314. Published 2018 May 2. doi:10.1186/s12864-018-4699-5

32.An ZX, Shi LG, Hou GY, Zhou HL, Xun WJ. Genetic diversity and selection signatures in Hainan black goats revealed by whole-genome sequencing data. Animal. 2024 Jun;18(6):101147. doi: 10.1016/j.animal.2024.101147.

33.Zeng M, Wang B, Liu L, Yang Y, Tang Z. Genome-wide association study identifies 12 new genetic loci associated with growth traits in pigs. Journal of Integrative Agriculture. 2024 Jan 1;23(1):217-27.

34.Zhao F, Xie R, Fang L, Xiang R, Yuan Z, Liu Y, Wang L. Analysis of 206 whole-genome resequencing reveals selection signatures associated with breed-specific traits in Hu sheep. Evol Appl. 2024 Jun 21;17(6):e13697. doi: 10.1111/eva.13697."

35.Zhao F, Xie R, Fang L, Xiang R, Yuan Z, Liu Y, Wang L. Analysis of 206 whole-genome resequencing reveals selection signatures associated with breed-specific traits in Hu sheep. Evol Appl. 2024 Jun 21;17(6):e13697. doi: 10.1111/eva.13697.

36.González-Prendes R, Quintanilla R, Mármol-Sánchez E, Pena RN, Ballester M, Cardoso TF, Manunza A, Casellas J, Cánovas Á, Díaz I, Noguera JL, Castelló A, Mercadé A, Amills M. Comparing the mRNA expression profile and the genetic determinism of intramuscular fat traits in the porcine gluteus medius and longissimus dorsi muscles. BMC Genomics. 2019 Mar 4;20(1):170. doi: 10.1186/s12864-019-5557-9.

37.Di WU, Qian XI, Xin-yun LI, Shu-hong ZH. Inducible overexpression of porcine homeobox A10 in the endometrium of transgenic mice. Journal of Integrative Agriculture. 2016 Jun 1;15(6):1338-44.

38.Niu Y, Li Y, Zhao Y, He X, Zhao Q, Pu Y, Ma Y, Jiang L. Whole-genome sequencing identifies functional genes for environmental adaptation in Chinese sheep. Journal of Genetics and Genomics. 2024 Nov 1;51(11):1278-85.

39.Wang X, Wang L, Shi L, Zhang P, Li Y, Li M, Tian J, Wang L, Zhao F. GWAS of reproductive traits in large white pigs on chip and imputed whole-genome sequencing data. International Journal of Molecular Sciences. 2022 Nov 1;23(21):13338.

40.Haque MA, Alam MZ, Iqbal A, Lee YM, Dang CG, Kim JJ. Genome-wide Association studies for body conformation traits in Korean Holstein Population. Animals. 2023 Sep 19;13(18):2964.

41.Purfield DC, Bradley DG, Evans RD, Kearney FJ, Berry DP. Genome-wide association study for calving performance using high-density genotypes in dairy and beef cattle. Genet Sel Evol. 2015 Jun 12;47(1):47. doi: 10.1186/s12711-015-0126-4.

42.Zhang S, Gao X, Jiang Y, Shen Y, Xie H, Pan P, Huang Y, Wei Y, Jiang Q. Population validation of reproductive gene mutation loci and association with the litter size in Nubian

goat. Arch Anim Breed. 2021 Sep 17;64(2):375-386. doi: 10.5194/aab-64-375-2021.
43.Zhou P, Yin C, Wang Y, Yin Z, Liu Y. Genomic Association Analysis of Growth and Backfat Traits in Large White Pigs. Genes (Basel). 2023 Jun 13;14(6):1258. doi: 10.3390/genes14061258.
44.Salatta BM, Muniz MMM, Fonseca LFS, Mota LFM, de Souza Teixeira C, Frezarim GB, Serna-García M, Dos Santos Silva DB, Pereira ASC, Baldi F, de Albuquerque LG. Skeletal muscle lncRNA profile associated with fatty acids in Nellore beef cattle. Sci Rep. 2025 Jul 18;15(1):26109. doi: 10.1038/s41598-025-11179-4.
45.Xie X, Huang C, Huang Y, Zou X, Zhou R, Ai H, Huang L, Ma J. Genetic architecture for skeletal muscle glycolytic potential in Chinese Erhualian pigs revealed by a genome-wide association study using 1.4M SNP array. Front Genet. 2023 Mar 17;14:1141411. doi: 10.3389/fgene.2023.1141411.
46.Silva MRGD, Veroneze R, Marques DBD, Silva DAD, Machado II, Brito LF, Lopes PS. A meta-analysis of genome-wide association studies to identify candidate genes associated with feed efficiency traits in pigs. J Anim Sci. 2025 Jan 4;103:skaf010. doi: 10.1093/jas/skaf010.
47.Liu J, Xu L, Ding X, Ma Y. Genome-Wide Association Analysis of Reproductive Traits in Chinese Holstein Cattle. Genes (Basel). 2023 Dec 20;15(1):12. doi: 10.3390/genes15010012.
48.Fang L, Cai W, Liu S, Canela-Xandri O, Gao Y, Jiang J, Rawlik K, Li B, Schroeder SG, Rosen BD, Li CJ, Sonstegard TS, Alexander LJ, Van Tassell CP, VanRaden PM, Cole JB, Yu Y, Zhang S, Tenesa A, Ma L, Liu GE. Comprehensive analyses of 723 transcriptomes enhance genetic and biological interpretations for complex traits in cattle. Genome Res. 2020 May;30(5):790-801. doi: 10.1101/gr.250704.119. Epub 2020 May 18.
49.Lan Y, Yan D, Li X, Zhou C, Bai Y, Dong X. Muscle growth differences in Lijiang pigs revealed by ATAC-seq multi-omics. Front Vet Sci. 2024 Aug 26;11:1431248. doi: 10.3389/fvets.2024.1431248.
50.Chang L, Zheng Y, Li S, et al. Identification of genomic characteristics and selective signals in Guizhou black goat. BMC Genomics. 2024;25(1):164. Published 2024 Feb 9. doi:10.1186/s12864-023-09954-6
51.Chang L, Zheng Y, Li S, Niu X, Huang S, Long Q, Ran X, Wang J. Identification of genomic characteristics and selective signals in Guizhou black goat. BMC Genomics. 2024 Feb 9;25(1):164. doi: 10.1186/s12864-023-09954-6.
52.Gu H, Zhu Q, Li Y, et al. Association Between InDel and CNV Variation in the FBLN1 Gene and Slaughter Traits in Cattle. Agriculture. 2025;15(5):518. doi:https://doi.org/10.3390/agriculture15050518
53.Bai F, Cai Y, Qiu M, et al. LCORL and STC2 variants increase body size and growth rate in cattle and other animals. bioRxiv. 2025. doi:https://doi.org/10.1101/2025.01.01.630985
54.Yang D, Zhang M, Chen W, Lu Q, Wan S, Du X, Li Y, Li B, Wu W, Wang C, Li N, Peng S, Tang H, Hua J. UCHL1 maintains microenvironmental homeostasis in goat germline stem cells. FASEB J. 2023 Dec;37(12):e23306. doi: 10.1096/fj.202301674RR.

55.Hua R, Zhou L, Zhang H, Yang H, Peng W, Wu K. Studying the variations in differently expressed serum proteins of Hainan black goat during the breeding cycle using isobaric tags for relative and absolute quantitation (iTRAQ) technology. J Reprod Dev. 2019 Oct 23;65(5):413-421. doi: 10.1262/jrd.2018-105. Epub 2019 Jul 14.
56.Xiao C, Jin HG, Zhang LC, Liu JQ, He M, Ma HH, Yu YS, Cao Y. Effects of SPARCL1 on the proliferation and differentiation of sheep preadipocytes. Adipocyte. 2021 Dec;10(1):658-669. doi: 10.1080/21623945.2021.2010901.
57.Ma Z, Chang Y, Brito LF, Li Y, Yang T, Wang Y, Yang N. Multitrait meta-analyses identify potential candidate genes for growth-related traits in Holstein heifers. J Dairy Sci. 2023 Dec;106(12):9055-9070. doi: 10.3168/jds.2023-23462.
58.Bakoev S, Getmantseva L, Kolosova M, Bakoev F, Kolosov A, Romanets E, Shevtsova V, Romanets T, Kolosov Y, Usatov A. Identifying Significant SNPs of the Total Number of Piglets Born and Their Relationship with Leg Bumps in Pigs. Biology (Basel). 2024 Dec 11;13(12):1034. doi: 10.3390/biology13121034.
59.Jiang J, Cao Y, Shan H, Wu J, Song X, Jiang Y. The GWAS Analysis of Body Size and Population Verification of Related SNPs in Hu Sheep. Front Genet. 2021 May 20;12:642552. doi: 10.3389/fgene.2021.642552.
60.Nazar M, Abdalla IM, Chen Z, Ullah N, Liang Y, Chu S, Xu T, Mao Y, Yang Z, Lu X. Genome-Wide Association Study for Udder Conformation Traits in Chinese Holstein Cattle. Animals (Basel). 2022 Sep 22;12(19):2542. doi: 10.3390/ani12192542."
61.Huang N, Zhao L, Wang J, Jiang Q, Ju Z, Wang X, Yang C, Gao Y, Wei X, Zhang Y, Xiao Y, Liu W, Lu S, Huang J. Signatures of selection in indigenous Chinese cattle genomes reveal adaptive genes and genetic variations to cold climate. J Anim Sci. 2023 Jan 3;101:skad006. doi: 10.1093/jas/skad006.
62.Zhu H, Li X, Zhang M, Liu S, Zhang Y, Zheng Y, Wei Z, Han M, Huang H, Fu T, Liang D. Detection of Selection Signatures and Genome-Wide Association Analysis of Body Weight Traits in Xianan Cattle. Genes (Basel). 2025 May 30;16(6):682. doi: 10.3390/genes16060682.
63.Bomfim GF, Merighe GKF, de Oliveira SA, Negrao JA. Acute and chronic effects of cortisol on milk yield, the expression of key receptors, and apoptosis of mammary epithelial cells in Saanen goats. J Dairy Sci. 2022;105(1):818-830. doi:10.3168/jds.2021-20364
64.Han M, Wang X, Du H, Cao Y, Zhao Z, Niu S, Bao X, Rong Y, Ao X, Guo F, Xia Q, Shang F, Wang R, Zhang Y. Genome-wide association study identifies candidate genes affecting body conformation traits of Zhongwei goat. BMC Genomics. 2025 Jan 14;26(1):37. doi: 10.1186/s12864-024-11097-1.
65.Sun Z, Liu Y, He X, Di R, Wang X, Ren C, Zhang Z, Chu M. Integrative Proteomics and Transcriptomics Profiles of the Oviduct Reveal the Prolificacy-Related Candidate Biomarkers of Goats (Capra hircus) in Estrous Periods. Int J Mol Sci. 2022 Nov 28;23(23):14888. doi: 10.3390/ijms232314888.
66.Reynolds EGM, Lopdell T, Wang Y, Tiplady KM, Harland CS, Johnson TJJ, Neeley C, Carnie K, Sherlock RG, Couldrey C, Davis SR, Harris BL, Spelman RJ, Garrick DJ, Littlejohn MD. Non-additive QTL mapping of lactation traits in 124,000 cattle reveals novel recessive loci. Genet Sel Evol. 2022 Jan 24;54(1):5. doi: 10.1186/s12711-021-00694-3.
67.Meng G, Bao Q, Ma X, Chu M, Huang C, Guo X, Liang C, Yan P. Analysis of Copy Number Variation in the Whole Genome of Normal-Haired and Long-Haired Tianzhu White

Yaks. Genes (Basel). 2022 Dec 18;13(12):2405. doi: 10.3390/genes13122405.
68.Crispim AC, Kelly MJ, Guimarães SE, et al. Multi-Trait GWAS and New Candidate Genes Annotation for Growth Curve Parameters in Brahman Cattle. PLoS One. 2015;10(10):e0139906. Published 2015 Oct 7. doi:10.1371/journal.pone.0139906
69.Nazar M, Lu X, Abdalla IM, Ullah N, Fan Y, Chen Z, Arbab AAI, Mao Y, Yang Z. Genome-Wide Association Study Candidate Genes on Mammary System-Related Teat-Shape Conformation Traits in Chinese Holstein Cattle. Genes (Basel). 2021 Dec 19;12(12):2020. doi: 10.3390/genes12122020.
70.Qiu J, Ma Z, Hong Z, Yin X, Chen Y, Ahmed HQ, Zan L, Li A. Comparative analysis of the whole transcriptome landscapes of muscle and adipose tissue in Qinchuan beef cattle. BMC Genomics. 2025 Jan 14;26(1):32. doi: 10.1186/s12864-025-11223-7.
71.Wang JJ, Li ZD, Zheng LQ, Zhang T, Shen W, Lei CZ. Genome-wide detection of selective signals for fecundity traits in goats (Capra hircus). Gene. 2022;818:146221. doi:10.1016/j.gene.2022.146221
72.Li, J., Sun, L., Sun, J., & Jiang, H. (2024). Genome-wide association research on the reproductive traits of Qianhua Mutton Merino sheep. Animal Bioscience, 37(9), 1535.
73.Hao Z, Jin X, Wang J, Luo Y, Hu J, Liu X, Li S, Zhao F, Li M. Functional differentiation of the ovine preadipocytes -insights from gene expression profiling. Funct Integr Genomics. 2023 Mar 23;23(2):97. doi: 10.1007/s10142-023-01034-y."
74.Reyes GC, Innes DJ, Ellis JL, Fox MK, Cant JP. Relationship between rate of glucose or propionate infusion and milk protein yield and concentration in dairy cows: A meta-regression. J Dairy Sci. 2024;107(5):2785-2796. doi:10.3168/jds.2023-23644
75.Yi KangLe, Yi KangLe, Li Chun, Qin QiLi Qin QiLi, Chen Lu Chen Lu, Sun YanLing Sun YanLing, Chen HuaHai Chen HuaHai, Yan HaiFeng Yan HaiFeng, Shi DeShun Shi DeShun, and Zhou Xu Zhou Xu. cDNA cloning and expression of the bovine factor in the germline alpha (FIGLA) gene in oocytes and ovarian follicles. (2011): 260-270.
76.Pausch H, Emmerling R, Schwarzenbacher H, Fries R. A multi-trait meta-analysis with imputed sequence variants reveals twelve QTL for mammary gland morphology in Fleckvieh cattle. Genet Sel Evol. 2016 Feb 16;48:14. doi: 10.1186/s12711-016-0190-4.
77.Piles M, Fernandez-Lozano C, Velasco-Galilea M, González-Rodríguez O, Sánchez JP, Torrallardona D, Ballester M, Quintanilla R. Machine learning applied to transcriptomic data to identify genes associated with feed efficiency in pigs. Genet Sel Evol. 2019 Mar 13;51(1):10. doi: 10.1186/s12711-019-0453-y.
78.Wang J, Shen N, Zhao K, Liao J, Jiang G, Xiao J, Jia X, Sun W, Lai S. Revealing study and breeding implications for production traits and tail characteristics in Simmental cattle by GWAS. Front Genet. 2025 Jan 31;16:1491816. doi: 10.3389/fgene.2025.1491816.
79.Zheng B, Hu X, Hu Y, Dong S, Xiao X, Qi H, Wang Y, Wang W, Wang Z. Type III adenylyl cyclase is essential for follicular development in female mice and their reproductive lifespan. iScience. 2024 Jun 17;27(7):110293. doi: 10.1016/j.isci.2024.110293.
80.Ma L, Luo H, Brito LF, Chang Y, Chen Z, Lou W, Zhang F, Wang L, Guo G, Wang Y. Estimation of genetic parameters and single-step genome-wide association studies for milk urea nitrogen in Holstein cattle. J Dairy Sci. 2023 Jan;106(1):352-363. doi: 10.3168/jds.2022-21857.

81.Ooi E, Xiang R, Chamberlain AJ, Goddard ME. Archetypal clustering reveals physiological mechanisms linking milk yield and fertility in dairy cattle. J Dairy Sci. 2024 Jul;107(7):4726-4742. doi: 10.3168/jds.2023-23699.

82.Eusebi PG, González-Prendes R, Quintanilla R, Tibau J, Cardoso TF, Clop A, Amills M. A genome-wide association analysis for carcass traits in a commercial Duroc pig population. Anim Genet. 2017 Aug;48(4):466-469. doi: 10.1111/age.12545.

83.Chen Y, Li R, Sun J, Li C, Xiao H, Chen S. Genome-Wide Population Structure and Selection Signatures of Yunling Goat Based on RAD-seq. Animals (Basel). 2022 Sep 13;12(18):2401. doi: 10.3390/ani12182401.

84.Zhang C, Asadollahpour Nanaei H, Jafarpour Negari N, Amiri Roudbar M, Amiri Ghanatsaman Z, Niyazbekova Z, Yang X. Genomic analysis uncovers novel candidate genes related to adaptation to tropical climates and milk production traits in native goats. BMC Genomics. 2024 May 14;25(1):477. doi: 10.1186/s12864-024-10387-y."

85.Zhao F, Xie R, Fang L, Xiang R, Yuan Z, Liu Y, Wang L. Analysis of 206 whole-genome resequencing reveals selection signatures associated with breed-specific traits in Hu sheep. Evol Appl. 2024 Jun 21;17(6):e13697. doi: 10.1111/eva.13697.

86.Zhang Y, Li F, Feng X, Yang H, Zhu A, Pang J, Han L, Zhang T, Yao X, Wang F. Genome-wide analysis of DNA Methylation profiles on sheep ovaries associated with prolificacy using whole-genome Bisulfite sequencing. BMC Genomics. 2017 Oct 2;18(1):759. doi: 10.1186/s12864-017-4068-9.

87.Salvetti NR, Alfaro NS, Velázquez MM, et al. Alteration in localization of steroid hormone receptors and coregulatory proteins in follicles from cows with induced ovarian follicular cysts. Reproduction. 2012;144(6):723-735. doi:10.1530/REP-12-0188

88.Deng K, Liu Z, Su Y, Zhang Z, Fan Y, Zhang Y, Wang F. RUNX1T1 modulates myogenic differentiation by regulating the calcium signaling pathway and the alternative splicing of ROCK2. FASEB J. 2023 Jul;37(7):e23044. doi: 10.1096/fj.202300677R.

89.Ashja A, Zorc M, Dovc P. Genome-Wide Association Study for Milk Somatic Cell Score in Holstein Friesian Cows in Slovenia. Animals (Basel). 2024;14(18):2713. Published 2024 Sep 19. doi:10.3390/ani14182713

90.Liu Y, Guo Z, Ni J, et al. Identification of Genetic Markers of APOM and CYP7A1 Genes Affecting Milk Production Traits in Chinese Holstein. Agriculture. 2025;15(1):33. doi:https://doi.org/10.3390/agriculture15010033

91.Bohlouli, M., Halli, K., Yin, T., Gengler, N., & König, S. (2022). Genome-wide associations for heat stress response suggest potential candidate genes underlying milk fatty acid composition in dairy cattle. Journal of Dairy Science, 105(4), 3323-3340.

92.Luo H, Li X, Hu L, Xu W, Chu Q, Liu A, Guo G, Liu L, Brito LF, Wang Y. Genomic analyses and biological validation of candidate genes for rectal temperature as an indicator of heat stress in Holstein cattle. J Dairy Sci. 2021 Apr;104(4):4441-4451. doi: 10.3168/jds.2020-18725. Epub 2021 Feb 13. Erratum in: J Dairy Sci. 2021 Jul;104(7):8338. doi: 10.3168/jds.2021-104-7-8338."

93.Tao L, He X, Wang X, Di R, Chu M. Litter Size of Sheep (Ovis aries): Inbreeding Depression and Homozygous Regions. Genes (Basel). 2021 Jan 18;12(1):109. doi:

10.3390/genes12010109.
94.Zhong, T., Wu, Y., Wang, Y., Lei, A., Wang, X., Hou, D., ... & Niu, L. (2025). Effects of early weaning on the development of gastrointestinal tract, rumen microbiota and host gene regulation in goat kids. Small Ruminant Research, 242, 107409.
95.Cheruiyot EK, Haile-Mariam M, Cocks BG, MacLeod IM, Xiang R, Pryce JE. New loci and neuronal pathways for resilience to heat stress in cattle. Sci Rep. 2021 Aug 17;11(1):16619. doi: 10.1038/s41598-021-95816-8. PMID: 34404823; PMCID: PMC8371109."
96.Plemyashov K, Krutikova A, Belikova A, Kuznetsova T, Semenov B. Genome-Wide Association Study (GWAS) for Left Displaced Abomasum in Highly Productive Russian Holstein Cattle. Animals (Basel). 2024;14(19):2795. Published 2024 Sep 27. doi:10.3390/ani14192795
97.Yang D, Jiang T, Liu J, et al. CREB3 regulatory factor -mTOR-autophagy regulates goat endometrial function during early pregnancy. Biol Reprod. 2018;98(5):713-721. doi:10.1093/biolre/ioy044
98.Caraballo LAS, Filho LASF, Sena LS, Biagiotti D, de Moura JDS, de Sousa Júnior A, Rocha AO, de Sousa FCB, da Silva Santos NP, Sarmento JLR. Genome-wide association study applied to prolificacy in Santa Inês sheep. Trop Anim Health Prod. 2025 Apr 11;57(3):169. doi: 10.1007/s11250-025-04424-5.
99.Piersanti RL, Horlock AD, Block J, Santos JEP, Sheldon IM, Bromfield JJ. Persistent effects on bovine granulosa cell transcriptome after resolution of uterine disease. Reproduction. 2019 Jul;158(1):35-46. doi: 10.1530/REP-19-0037.
100.Pausch H, Emmerling R, Schwarzenbacher H, Fries R. A multi-trait meta-analysis with imputed sequence variants reveals twelve QTL for mammary gland morphology in Fleckvieh cattle. Genet Sel Evol. 2016 Feb 16;48:14. doi: 10.1186/s12711-016-0190-4.
101.de Lima AO, Koltes JE, Diniz WJS, et al. Potential Biomarkers for Feed Efficiency-Related Traits in Nelore Cattle Identified by Co-expression Network and Integrative Genomics Analyses. Front Genet. 2020;11:189. Published 2020 Mar 4. doi:10.3389/fgene.2020.00189
102.Yuan Y, Zhang WY, Yang BG, et al. A 1.1 Mb duplication CNV on chromosome 17 contributes to skeletal muscle development in Boer goats. Zool Res. 2023;44(2):303-314. doi:10.24272/j.issn.2095-8137.2022.384
103.Luo H, Hu L, Brito LF, et al. Weighted single-step GWAS and RNA sequencing reveals key candidate genes associated with physiological indicators of heat stress in Holstein cattle. J Anim Sci Biotechnol. 2022;13(1):108. Published 2022 Aug 20. doi:10.1186/s40104-022-00748-6
104.Wang J, Xie W, Li N, Li W, Zhang Z, Fan N, Ouyang Z, Zhao Y, Lai C, Li H, Chen M, Quan L, Li Y, Jiang Y, Jia W, Fu L, Mazid MA, Zhu Y, Maxwell PH, Pan G, Esteban MA, Dai Z, Lai L. Generation of a humanized mesonephros in pigs from induced pluripotent stem cells via embryo complementation. Cell Stem Cell. 2023 Sep 7;30(9):1235-1245.e6. doi: 10.1016/j.stem.2023.08.003.
105.Zhu X, Wei Y, Zhan Q, Yan A, Feng J, Liu L, Tang D. CRISPR/Cas9-Mediated Biallelic Knockout of IRX3 Reduces the Production and Survival of Somatic Cell-Cloned Bama Minipigs. Animals (Basel). 2020 Mar 17;10(3):501. doi: 10.3390/ani10030501.

106.Sheriff O, Ahbara AM, Haile A, Alemayehu K, Han JL, Mwacharo JM. Whole-genome resequencing reveals genomic variation and dynamics in Ethiopian indigenous goats. Front Genet. 2024;15:1353026. Published 2024 May 24. doi:10.3389/fgene.2024.1353026

107.Chen Q, Zhang F, Qu K, et al. Genome-wide association study identifies genomic loci associated with flight reaction in cattle. J Anim Breed Genet. 2020;137(5):477-485. doi:10.1111/jbg.12461

108.Ooi E, Xiang R, Chamberlain AJ, Goddard ME. Archetypal clustering reveals physiological mechanisms linking milk yield and fertility in dairy cattle. J Dairy Sci. 2024 Jul;107(7):4726-4742. doi: 10.3168/jds.2023-23699.

109.Gholizadeh M, Esmaeili-Fard SM. Meta-analysis of genome-wide association studies for litter size in sheep. Theriogenology. 2022 Mar 1;180:103-112. doi: 10.1016/j.theriogenology.2021.12.025.

110.Martinez-Royo A, Alabart JL, Sarto P, Serrano M, Lahoz B, Folch J, Calvo JH. Genome-wide association studies for reproductive seasonality traits in Rasa Aragonesa sheep breed. Theriogenology. 2017 Sep 1;99:21-29. doi: 10.1016/j.theriogenology.2017.05.011."

111.Rong Y, Ao X, Guo F, Wang X, Han M, Zhang L, Xia Q, Shang F, Lv Q, Wang Z, Su R, Zhao Y, Zhang Y, Wang R. Genome-Wide Association Analysis Revealed Candidate Genes Related to Early Growth Traits in Inner Mongolia Cashmere Goats. Vet Sci. 2025 Feb 20;12(3):192. doi: 10.3390/vetsci12030192.

112.Constantino JV, Carranza-Martin A, Premanandan C, Kirkpatrick BW, Wiltbank MC, Garcia-Guerra A. Preantral follicle numbers and size in heifers carrying Trio, a bovine high fecundity allele. Reproduction. 2023 May 25;166(1):13-26. doi: 10.1530/REP-23-0072.

113.Tao L, He XY, Wang FY, Pan LX, Wang XY, Gan SQ, Di R, Chu MX. Identification of genes associated with litter size combining genomic approaches in Luzhong mutton sheep. Anim Genet. 2021 Aug;52(4):545-549. doi: 10.1111/age.13078.

114.Yu H, Yu S, Guo J, Cheng G, Mei C, Zan L. Genome-Wide Association Study Reveals Novel Loci Associated with Body Conformation Traits in Qinchuan Cattle. Animals (Basel). 2023 Nov 23;13(23):3628. doi: 10.3390/ani13233628.

115.Tao L, He XY, Wang FY, Pan LX, Wang XY, Gan SQ, Di R, Chu MX. Identification of genes associated with litter size combining genomic approaches in Luzhong mutton sheep. Anim Genet. 2021 Aug;52(4):545-549. doi: 10.1111/age.13078.

116.Zhou Y, Ren W, Shao W, Gao Y, Yao K, Yang M, Zhang X, Wang Y, Li F, Yang L. Exploration of non-coding RNAs related to intramuscular fat deposition Xinjiang Brown cattle and Angus × Wagyu cattle. BMC Genomics. 2025 Mar 14;26(1):249. doi: 10.1186/s12864-025-11453-9."

117.Liang C, Han M, Zhou Z, Liu Y, He X, Jiang Y, Ouyang Y, Hong Q, Chu M. Hypothalamic Transcriptome Analysis Reveals the Crucial MicroRNAs and mRNAs Affecting Litter Size in Goats. Front Vet Sci. 2021 Nov 1;8:747100. doi: 10.3389/fvets.2021.747100.

118.Zhang D, Yu H, Gu M, Zhang S, Ma X, Zhang W, Zhu Y, Al-Wraikat M, Abubaker MA, Zhang R, Liu Y. Unveils key proteins in Xinjiang goat muscle linked to post-mortem meat quality: A TMT-based proteomic analysis. Food Chem X. 2024 Sep 20;24:101847. doi: 10.1016/j.fochx.2024.101847."

119.Ma X, Cheng H, Liu Y, Sun L, Chen N, Jiang F, You W, Yang Z, Zhang B, Song E, Lei C. Assessing Genomic Diversity and Selective Pressures in Bohai Black Cattle Using Whole-Genome Sequencing Data. Animals (Basel). 2022 Mar 7;12(5):665. doi: 10.3390/ani12050665.

120.Lv W, An R, Li X, Zhang Z, Geri W, Xiong X, Yin S, Fu W, Liu W, Lin Y, Li J, Xiong Y. Multi-Omics Approaches Uncovered Critical mRNA-miRNA-lncRNA Networks Regulating Multiple Birth Traits in Goat Ovaries. Int J Mol Sci. 2024 Nov 20;25(22):12466. doi: 10.3390/ijms252212466.

121.Marrella M, Schettini GP, Morozyuk M, Walsh A, Cockrum R, Biase F. Genome-wide association analyses in dairy heifers highlight genes overlapping with mouse and human fertility and human health traits. bioRxiv. 2024. doi:https://doi.org/10.1101/2024.12.19.629410

122.Dias MS, Pedrosa VB, Rocha da Cruz VA, Silva MR, Batista Pinto LF. Genome-wide association and functional annotation analysis for the calving interval in Nellore cattle. Theriogenology. 2024 Apr 1;218:214-222. doi: 10.1016/j.theriogenology.2024.01.034.

123.Li R, Li C, Chen H, et al. Genome-wide scan of selection signatures in Dehong humped cattle for heat tolerance and disease resistance. Anim Genet. 2020;51(2):292-299. doi:10.1111/age.12896

124.Wang L, Wang Y, Li B, et al. BMP6 regulates AMH expression via SMAD1/5/8 in goat ovarian granulosa cells. Theriogenology. 2023;197:167-176. doi:10.1016/j.theriogenology.2022.11.045

125.Yang X, Li Y, Sun B, et al. Effect of GnRHR, BMP6 and FSHR Gene Pyramiding on Litter Traits of Goats. Animals (Basel). 2025;15(10):1358. doi:10.3390/ani15101358"

126.Huang C, Zhao Q, Chen Q, Su Y, Ma Y, Ye S, Zhao Q. Runs of Homozygosity Detection and Selection Signature Analysis for Local Goat Breeds in Yunnan, China. Genes (Basel). 2024 Feb 28;15(3):313. doi: 10.3390/genes15030313.

127.Zhang Z, Kargo M, Liu A, Thomasen JR, Pan Y, Su G. Genotype-by-environment interaction of fertility traits in Danish Holstein cattle using a single-step genomic reaction norm model. Heredity (Edinb). 2019 Aug;123(2):202-214. doi: 10.1038/s41437-019-0192-4. Epub 2019 Feb 13.

128.Jiao S, Maltecca C, Gray KA, Cassady JP. Feed intake, average daily gain, feed efficiency, and real-time ultrasound traits in Duroc pigs: II. Genomewide association. J Anim Sci. 2014 Jul;92(7):2846-60. doi: 10.2527/jas.2014-7337.

129.Zhang L, Michal JJ, O'Fallon JV, Pan Z, Gaskins CT, Reeves JJ, Busboom JR, Zhou X, Ding B, Dodson MV, Jiang Z. Quantitative genomics of 30 complex phenotypes in Wagyu x Angus F_1 progeny. Int J Biol Sci. 2012;8(6):838-58. doi: 10.7150/ijbs.4403. Epub 2012 Jun 12.

130.Silva ÉF, Lopes MS, Lopes PS, Gasparino E. A genome-wide association study for feed efficiency-related traits in a crossbred pig population. Animal. 2019 Nov;13(11):2447-2456. doi: 10.1017/S1751731119000910. Epub 2019 May 28.

131.Gangwar M, Kumar S, Ahmad SF, Singh A, Agrawal S, Anitta PL, Kumar A. Identification of genetic variants affecting reproduction traits in Vrindavani cattle. Mamm Genome. 2024 Mar;35(1):99-111. doi: 10.1007/s00335-023-10023-2.

132.Mohammadi A, Alijani S, Seyed AR, Abdollahi-Arpanahi R. Genome-Wide Association Study and Pathway Analysis for Female Fertility Traits in Iranian Holstein Cattle. Annals

of Animal Science. 2020;20(3):825-851. doi:https://doi.org/10.2478/aoas-2020-0031

133.Zhao Z, Yang Y, Liu P, Yan T, Li R, Pan C, Li Y, Lan X. A Critical Functional Missense Mutation (T117M) in Sheep MC4R Gene Significantly Leads to Gain-of-Function. Animals (Basel). 2024 Jul 30;14(15):2207. doi: 10.3390/ani14152207.

134.Liu X, Li H, Wang L, Zhang L, Wang L. The Effect of Sow Maternal Behavior on the Growth of Piglets and a Genome-Wide Association Study. Animals (Basel). 2023 Dec 5;13(24):3753. doi: 10.3390/ani13243753.

135.Gualdrón Duarte JL, Yuan C, Gori AS, Moreira GCM, Takeda H, Coppieters W, Charlier C, Georges M, Druet T. Sequenced-based GWAS for linear classification traits in Belgian Blue beef cattle reveals new coding variants in genes regulating body size in mammals. Genet Sel Evol. 2023 Nov 28;55(1):83. doi: 10.1186/s12711-023-00857-4.

136.Pausch H, Emmerling R, Schwarzenbacher H, Fries R. A multi-trait meta-analysis with imputed sequence variants reveals twelve QTL for mammary gland morphology in Fleckvieh cattle. Genet Sel Evol. 2016 Feb 16;48:14. doi: 10.1186/s12711-016-0190-4."

137.Chang L, Zheng Y, Li S, Niu X, Huang S, Long Q, Ran X, Wang J. Identification of genomic characteristics and selective signals in Guizhou black goat. BMC Genomics. 2024 Feb 9;25(1):164. doi: 10.1186/s12864-023-09954-6.

138.Molinero E, Pena RN, Estany J, Ros-Freixedes R. Unravelling novel and closely linked association signals for fat-related traits in pigs using prioritised variants from whole-genome sequence data. Animal. 2025 May;19(5):101496. doi: 10.1016/j.animal.2025.101496.

139.Du Z, D'Alessandro E, Asare E, Zheng Y, Wang M, Chen C, Wang X, Song C. Retrotransposon Insertion Polymorphisms (RIPs) in Pig Reproductive Candidate Genes. Genes (Basel). 2022 Jul 28;13(8):1359. doi: 10.3390/genes13081359.

140. Wei Y, Zhu C, He X, Chu M. Hypothalamus Transcriptome Reveals Key lncRNAs and mRNAs Associated with Fecundity in Goats. Animals (Basel). 2025;15(5):754. Published 2025 Mar 6. doi:10.3390/ani15050754

141.An ZX, Shi LG, Hou GY, Zhou HL, Xun WJ. Genetic diversity and selection signatures in Hainan black goats revealed by whole-genome sequencing data. Animal. 2024;18(6):101147. doi:10.1016/j.animal.2024.101147"
