# Supplementary material for: Integrating selective signature analysis and genome-wide association study to identify candidate genes associated with litter size in Leizhou goat
Source: Anim Biosci. 2026 Jul 27;39(11):260280. doi: 10.5713/ab.260280 (PMC13740650; doi:10.5713/ab.260280)
Supplement: Supplementary file 12 [file ab-260280-Supplementary-6.pdf]

Supplement 6. Results of KEGG enrichment analysis for the 346 candidate genes identified by selection signature analysis.

Term	ID	Input number	Background number	*P*-Value	Input
FoxO signaling pathway	chx04068	9	130	0.000128	*TNFSF10*|*BCL2L11*|*CDKN2D*|*PRKAG2*|*CHUK*|*INSR*|*CCND2*|*MAPK8*|*EP300*
Pathways in cancer	chx05200	17	537	0.001716	*NCOA3*|*BCL2L11*|*ESR1*|*FZD9*|*ADCY3*|*CCND2*|*CHUK*|*CAMK2B*|*PTGER3*|*LAMC2*|*RUNX1T1*|*EDNRA*|*MTOR*|*LPAR3*|*MAPK8*|*EP300*|*KITLG*
Insulin secretion	chx04911	6	88	0.001838	*KCNMB4*|*ADCY3*|*CAMK2B*|*PCLO*|*KCNN2*|*RAB3A*
Longevity regulating pathway - multiple species	chx04213	5	62	0.002222	*RPTOR*|*INSR*|*PRKAG2*|*MTOR*|*ADCY3*
Adherens junction	chx04520	5	72	0.004072	*INSR*|*EP300*|*SSX2IP*|*NECTIN4*|*LMO7*
Type II diabetes mellitus	chx04930	4	46	0.00479	*MAPK8*|*MTOR*|*INSR*|*CACNA1E*
Axon guidance	chx04360	8	185	0.005175	*BMP7*|*EPHB1*|*SEMA3E*|*RYK*|*UNC5B*|*SRGAP1*|*CAMK2B*|*TRPC3*
Transcriptional misregulation in cancer	chx05202	8	190	0.006015	*TCF3*|*CCND2*|*HOXA11*|*HOXA10*|*ERG*|*RUNX1T1*|*DUSP6*|*HOXA9*
cAMP signaling pathway	chx04024	9	247	0.008881	*NPR1*|*ADCY3*|*GRIA4*|*PTGER3*|*CAMK2B*|*EDNRA*|*MAPK8*|*EP300*|*GHSR*
Longevity regulating pathway	chx04211	5	89	0.009387	*RPTOR*|*INSR*|*PRKAG2*|*MTOR*|*ADCY3*

Regulation of lipolysis in adipocytes	chx04923	4	59	0.010797	*NPR1|INSR|ADCY3|PTGER3*
Endocrine resistance	chx01522	5	93	0.011116	*NCOA3|MAPK8|ADCY3|MTOR|ESR1*
Autophagy - other	chx04136	3	34	0.013899	*RPTOR|MTOR|ATG4D*
Melanogenesis	chx04916	5	100	0.014644	*FZD9|KITLG|EP300|CAMK2B|ADCY3*
Vascular smooth muscle contraction	chx04270	6	140	0.015259	*NPR1|AVPR1A|KCNMB4|ADCY3|EDNRA|CALD1*
Insulin signaling pathway	chx04910	6	140	0.015259	*RPTOR|MKNK2|PRKAG2|INSR|MTOR|MAPK8*
Acute myeloid leukemia	chx05221	4	67	0.016188	*MTOR|DUSP6|RUNX1T1|CHUK*
Th17 cell differentiation	chx04659	5	105	0.017578	*MAPK8|MTOR|IL17F|IL17A|CHUK*
PI3K-Akt signaling pathway	chx04151	11	375	0.017636	*RPTOR|NGF|BCL2L11|CCND2|CHUK|PPP2R2C |INSR|MTOR|LAMC2|LPAR3|KITLG*
Phospholipase D signaling pathway	chx04072	6	148	0.01933	*AVPR1A|ADCY3|INSR|MTOR|LPAR3|KITLG*
Adipocytokine signaling pathway	chx04920	4	72	0.020282	*MAPK8|PRKAG2|MTOR|CHUK*
Apoptosis	chx04210	6	151	0.02103	*NGF|TNFSF10|BCL2L11|CHUK|SPTA1|MAPK8*
HIF-1 signaling pathway	chx04066	5	114	0.02379	*MKNK2|INSR|EP300|MTOR|CAMK2B*
mTOR signaling pathway	chx04150	6	156	0.024084	*RPTOR|FZD9|ATP6V1E1|CHUK|INSR|MTOR*

Calcium signaling pathway	chx04020	7	202	0.025115	*AVPR1A|ADCY3|CACNA1E|CAMK2B|PTGER3|SLC8A1|EDNRA*
MAPK signaling pathway	chx04010	9	299	0.026277	*NGF|MKNK2|CDC25B|CHUK|CACNA1E|INSR|MAPK8|DUSP6|KITLG*
Wnt signaling pathway	chx04310	6	163	0.028839	*RYK|FZD9|CCND2|CAMK2B|MAPK8|EP300*
Ovarian steroidogenesis	chx04913	3	47	0.030821	*BMP6|INSR|ADCY3*
AMPK signaling pathway	chx04152	5	125	0.033106	*RPTOR|INSR|PRKAG2|MTOR|PPP2R2C*
Cell cycle	chx04110	5	126	0.03405	*ANAPC1|CDC25B|CCND2|EP300|CDKN2D*
cGMP-PKG signaling pathway	chx04022	6	173	0.036643	*NPR1|KCNMB4|ADCY3|INSR|SLC8A1|EDNRA*
Progesterone-mediated oocyte maturation	chx04914	4	89	0.038651	*ANAPC1|CDC25B|MAPK8|ADCY3*
Dopaminergic synapse	chx04728	5	131	0.039023	*MAPK8|PPP2R2C|GRIA4|CAMK2B|SCN1A*
IL-17 signaling pathway	chx04657	4	90	0.039951	*MAPK8|IL17F|IL17A|CHUK*
Prostate cancer	chx05215	4	94	0.045401	*EP300|MTOR|CHUK|ERG*
Human T-cell leukemia virus 1 infection	chx05166	7	232	0.046437	*TCF3|ADCY3|CCND2|CHUK|ANAPC1|MAPK8|EP300*
TGF-beta signaling pathway	chx04350	4	96	0.048273	*BMP7|BMP6|EP300|NOG*

Glycosaminoglycan biosynthesis - heparan sulfate / heparin	chx00534	2	24	0.048684	*GLCE*|*NDST4*
