# Supplementary material for: Integrating selective signature analysis and genome-wide association study to identify candidate genes associated with litter size in Leizhou goat
Source: Anim Biosci. 2026 Jul 27;39(11):260280. doi: 10.5713/ab.260280 (PMC13740650; doi:10.5713/ab.260280)
Supplement: Supplementary file 13 [file ab-260280-Supplementary-7.pdf]

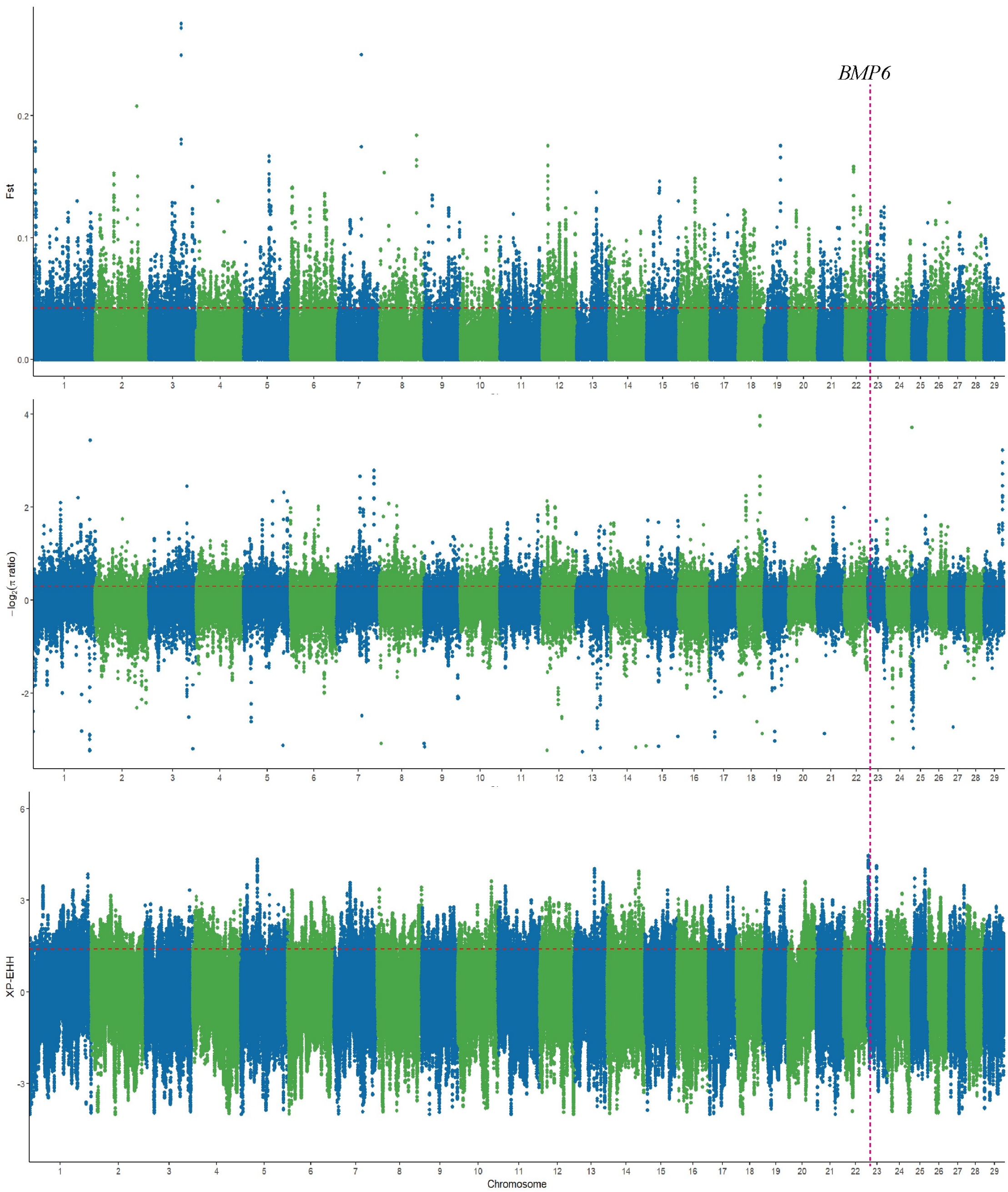


**Supplement 7.** Selective sweep analysis by comparing genomes between high and low litter size Leizhou goats. Pairwise fixation index ($F_{ST}$) (top panel), log2π-ratio (middle panel) and normalized XP-EHH scores (bottom panel) calculated between high and low litter size Leizhou goats in a 20 kb sliding window with a 5 kb step across all autosomes. The dashed horizontal lines indicate the significance threshold ($F_{ST}$ = 0.03, $\log_2\pi$-ratio = 0.3, XP-EHH = 1.41) used for extracting outliers.
