# Supplementary material for: Integrating selective signature analysis and genome-wide association study to identify candidate genes associated with litter size in Leizhou goat
Source: Anim Biosci. 2026 Jul 27;39(11):260280. doi: 10.5713/ab.260280 (PMC13740650; doi:10.5713/ab.260280)
Supplement: Supplementary file 14 [file ab-260280-Supplementary-8.pdf]

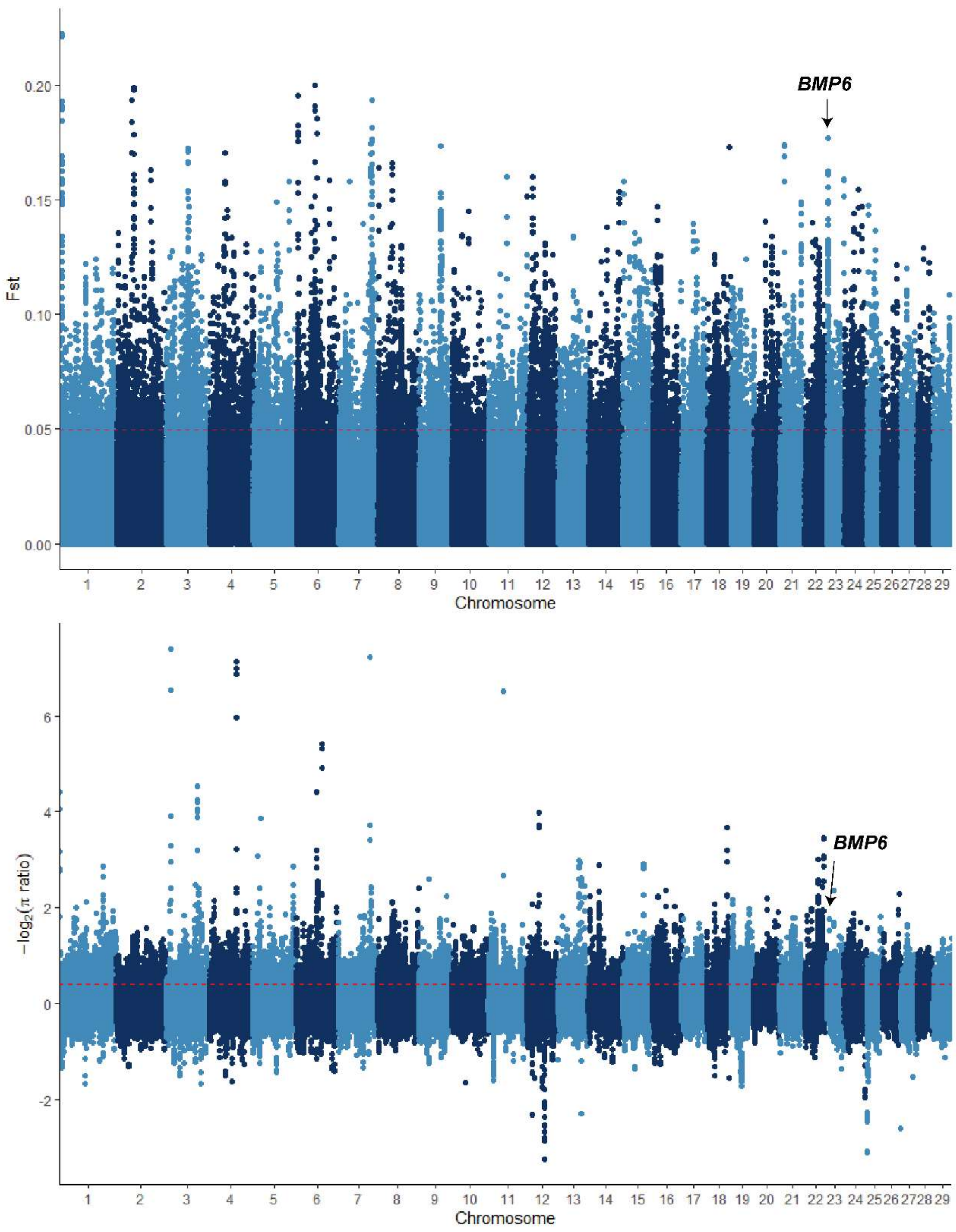

Supplement 8. Selective sweep analysis by comparing genomes between high (n=30) and low (n=62) litter size Leizhou goats. F_{ST}) (top panel), and log2π-ratio (bottom panel) calculated between high and low litter size Leizhou goats in a 20 kb sliding window with a 5 kb step across all autosomes. The dashed horizontal lines indicate the significance threshold (F_{ST} = 0.04, $\log_2\pi$-ratio = 0.413) used for extracting outliers.
