# Supplementary material for: Integrating selective signature analysis and genome-wide association study to identify candidate genes associated with litter size in Leizhou goat
Source: Anim Biosci. 2026 Jul 27;39(11):260280. doi: 10.5713/ab.260280 (PMC13740650; doi:10.5713/ab.260280)
Supplement: Supplementary file 15 [file ab-260280-Supplementary-9.pdf]

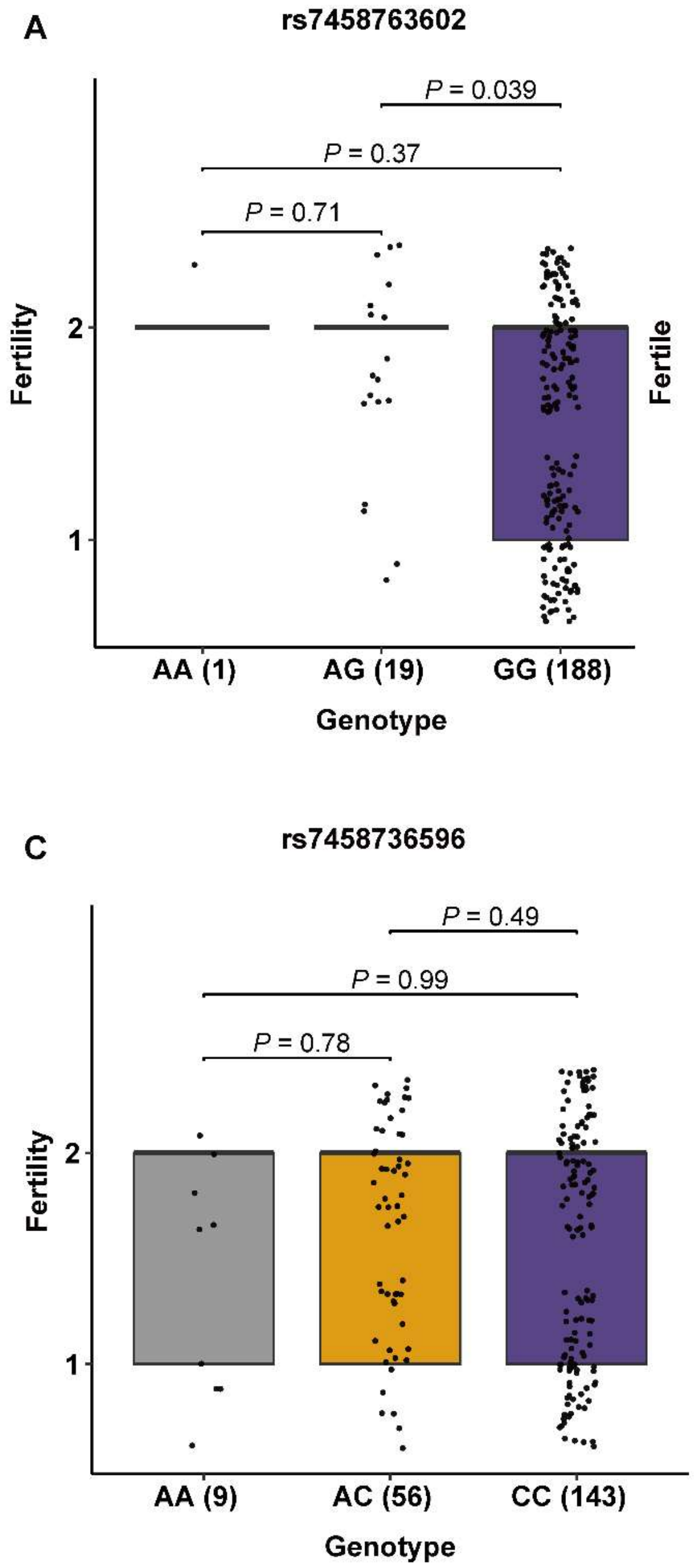

Supplement 9. The distribution of genotypes for the three key SNPs (A. rs7458763602. B. rs646562093. C. rs7458736596.) in the *BMP6* gene across high and low reproductive goat populations.
